# Giving structure to the biofilm matrix: an overview of individual strategies and emerging common themes

**DOI:** 10.1093/femsre/fuv015

**Published:** 2015-04-23

**Authors:** Laura Hobley, Catriona Harkins, Cait E. MacPhee, Nicola R. Stanley-Wall

**Affiliations:** 1 Division of Molecular Microbiology, College of Life Sciences, University of Dundee, Dundee DD1 5EH, UK; 2 James Clerk Maxwell Building, School of Physics, University of Edinburgh, Edinburgh EH9 3JZ, UK

**Keywords:** amyloid fibres, biophysics, biofilm matrix assembly, hydrophobin, *Bacillus subtilis*, *Escherichia coli*, *Vibrio cholerae*, *Staphylococcus aureus*

## Abstract

Biofilms are communities of microbial cells that underpin diverse processes including sewage bioremediation, plant growth promotion, chronic infections and industrial biofouling. The cells resident in the biofilm are encased within a self-produced exopolymeric matrix that commonly comprises lipids, proteins that frequently exhibit amyloid-like properties, eDNA and exopolysaccharides. This matrix fulfils a variety of functions for the community, from providing structural rigidity and protection from the external environment to controlling gene regulation and nutrient adsorption. Critical to the development of novel strategies to control biofilm infections, or the capability to capitalize on the power of biofilm formation for industrial and biotechnological uses, is an in-depth knowledge of the biofilm matrix. This is with respect to the structure of the individual components, the nature of the interactions between the molecules and the three-dimensional spatial organization. We highlight recent advances in the understanding of the structural and functional role that carbohydrates and proteins play within the biofilm matrix to provide three-dimensional architectural integrity and functionality to the biofilm community. We highlight, where relevant, experimental techniques that are allowing the boundaries of our understanding of the biofilm matrix to be extended using *Escherichia coli, Staphylococcus aureus, Vibrio cholerae*, and *Bacillus subtilis* as exemplars.

## Abstract (precis)

Examining the structure and function of the biofilm extracellular matrix.

## Graphical abstract

**Graphical Abstract Figure.**
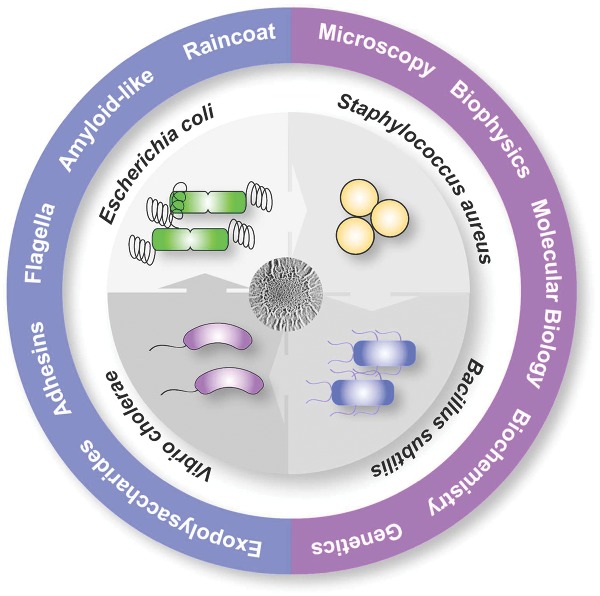
Examining the structure and function of the biofilm extracellular matrix.

## INTRODUCTION

A behaviour that connects microorganisms living in diverse environments is the formation of sessile social communities (Costerton *et al.* 1987). The word ‘biofilm’ was coined to describe these assemblages that are now widely regarded as the major reservoirs of bacteria and other microbes in the environment (Costerton *et al.* 1987). Biofilms have been identified as playing a role in beneficial interactions, including symbioses with other organisms, such as the formation of biofilms on plant tissues and the colonization of the squid by the luminescent bacteria *Aliivibrio fischeri* (formerly *Vibrio fischeri*) (Nyholm *et al.* 2000; Bais, Fall and Vivanco 2004; Yaron and Romling 2014). Conversely, biofilms have been implicated as being involved in many different chronic bacterial infections. For instance, the bacterial consortium that infects the lungs of people with the genetic disease cystic fibrosis is now recognized as being within a highly mucoid biofilm matrix, making them less susceptible to antibiotic treatment (Sherrard, Tunney and Elborn 2014). Moreover, the bacteria that cause infections in indwelling medical devices, such as catheters and artificial joint implants, are known to form biofilms during colonization of these devices (Hall, McGillicuddy and Kaplan 2014).

Biofilms are hallmarked by the production of an extracellular polymeric biofilm matrix (Costerton *et al.* 1987). This is not a recent finding as the fact that bacteria are capable of producing an extracellular material that aids attachment was recognized in the pre-molecular era by Claude Zobell and Esther Allen, who reported ‘The film of bacteria may promote the attachment of macroscopic organisms in different ways. They may form a mucilaginous surface to which the fouling organisms in the planktonic or free-swimming stage readily adhere until they can prepare their own holdfast’ (Zobell and Allen 1935). It is now known that the biofilm matrix produced by the majority of organisms commonly comprises eDNA, lipids, exopolysaccharides and extracellular proteins, many of which exhibit amyloid-like properties (Branda *et al.* 2005; Flemming and Wingender 2010). It is the production of the biofilm matrix that underpins the remarkable success of biofilm communities in allowing the propagation and survival of cells in their local environment (Davey and O'Toole 2000).

The function of the extracellular matrix within the biofilm is diverse and consistent with this it has a variable composition across diverse microbial species (Flemming and Wingender 2010). One strategy adopted by several species of bacteria to impart structural integrity/rigidity to the biofilm is the synthesis of protein fibres that form a scaffold onto which the cells and other matrix components (such as exopolysaccharides) are attached (Barnhart and Chapman 2006; Branda *et al.* 2006; Borlee *et al.* 2010). Other components in the matrix fulfil a protective function for the inhabitants. For instance, the bacterial hydrophobin BslA forms a water-resistant ‘raincoat’ over the *Bacillus subtilis* biofilm, and the cellulose produced by *Escherichia coli* biofilms increases the resistance of the community to desiccation (Gualdi *et al.* 2008; Kobayashi and Iwano 2012; Hobley *et al.* 2013). Further matrix components facilitate interactions between bacteria and host cells: for example, while curli fibres produced by *E. coli* form a structural component of the biofilm (Chapman *et al.* 2002; Serra *et al.* 2013b), they are also required for the attachment of the *E. coli* cells to a variety of protein components of the host cells at the onset of infection (Olsen, Jonsson and Normark 1989; Sjobring, Pohl and Olsen 1994; Ben Nasr *et al.* 1996). Detailed understanding of the molecular function of such components is critical to the ability to control initiation, stabilization or dispersal of biofilms.

Here we will discuss the molecular function of the exopolysaccharides, extracellular proteins and appendages (such as cell-wall-anchored proteins and flagella) that form the biofilm matrix. We focus on four bacterial species: *E. coli*, *B. subtilis*, *Staphylococcus aureus* and *Vibrio cholerae*, as the biofilm matrix composition and structure has been extensively studied for these organisms. We will examine the similarities between the underlying mechanisms deployed by these bacteria in building a structured community. At the same time, we highlight some of the different, and possibly unique, mechanisms that have evolved. We will also detail how recent advances in technology, in particular microscopy and spectroscopy tools, have allowed a more comprehensive analysis of the *in situ* components of the biofilm. Although comparatively new, the field of biofilm research is at an exciting point, as we move forward from the concept of biofilms simply consisting of a group of cells in an extracellular ‘slime’ to the understanding that the matrix is, in fact, a highly ordered structure that fulfils a great many roles for the bacteria resident within.

### ESCHERICHIA COLI

*Escherichia coli* is a Gram-negative bacterium in the family Enterobacteriaceae. Whilst it is commonly used as ‘the’ model bacterium in many laboratory studies, it is a commensal bacterium that resides in the gastrointestinal tract but has the potential to act as an opportunistic pathogen. It can cause a variety of infections in humans including diarrhoeal disease, urinary tract infections and sepsis/meningitis (for full reviews on *E. coli* pathogenesis, see Kaper, Nataro and Mobley 2004; Clements *et al.* 2012). Biofilm formation by *E. coli* has been extensively studied at the molecular level over the past 20 years. It can form biofilms on a variety of surfaces, including submerged biofilms on plastic and glass surfaces, macrocolony formation on agar plates, and floating pellicle biofilms at an air–liquid interface (Danese *et al.* 2000; Hung *et al.* 2013; Serra *et al.* 2013b). The vast array of genetic tools that are available to work with *E. coli* has facilitated many molecular genetics-based studies of biofilm formation. *E. coli* has been shown to produce a range of autotransporter adhesins, the most studied of which is Antigen 43 (Ag43), which acts to promote cell-to-cell adhesion and aggregation at the initial stages of biofilm formation. In the mature biofilm, the main conserved components of the *E. coli* biofilm matrix have been defined as the proteinaceous curli fibres and flagella, alongside the polysaccharide cellulose. Additional components of the biofilm matrix have been shown (in some strains) to include both β-1,6-*N*-acetyl-D-glucosamine (PGA) and colanic acid (Prigent-Combaret *et al.* 2000; Wang, Preston and Romeo 2004). Recent advances in microscopy techniques have elucidated the internal structure of the biofilm. Indeed using combinations of fluorescence light microscopy, transmission electron microscopy and scanning electron microscopy (SEM), it was found that these extracellular components and appendages provide structure to the biofilm and that they are found in discrete zones of the biofilm (Serra, Richter and Hengge 2013a; Serra *et al.* 2013b).

## AUTOTRANSPORTER ADHESINS

Cell-to-cell adhesion is mediated by a set of protein components made by *E. coli* that are termed autotransporter adhesins. A well-studied member of this class of proteins is the outer membrane protein Antigen 43 (Ag43 encoded by *agn43*). Antigen 43 has been shown to be responsible for aggregation of *E. coli* in stationary liquid cultures (Henderson, Meehan and Owen 1997; Hasman, Chakraborty and Klemm 1999). It was first shown to be important for biofilm formation in the *E. coli* strain W3110, where it is required for wild-type levels of submerged biofilm formation in glucose-minimal medium (Danese *et al.* 2000). An *agn43* deletion strain retained the ability to attach to PVC surfaces, but the biofilms were observed to be less dense than wild type when quantified using crystal-violet staining. The single *agn43* gene encodes for a protein that is processed into two separate subunits, the α- and β-subunits (Hasman, Chakraborty and Klemm 1999): the β-subunit is an integral outer membrane protein that is required for translocation of the α-subunit across the outer membrane, whilst the α-subunit is found on the cell surface, mediated through an interaction with the β-subunit. This autoaggregation of cells is dependent on Ag43–Ag43 interactions (Hasman, Chakraborty and Klemm 1999). Solution of the protein structure of Ag43 revealed the α-subunit to form an L-shaped protein, and that pairs of α-subunits use a combination of hydrogen bonds and salt bridges to stabilize intermicrobial protein dimers (Heras *et al.* 2014) which line up in a head-to-tail conformation, forming a protein ‘velcro’ that results in the autoaggregation of cells. Expression of *agn43*, and hence the production of Ag43, is under the control of phase variation, which means that cells are either in an *agn43* OFF or ON state (Schembri *et al.* 2003; Chauhan *et al.* 2013). Biofilm formation (as studied in the laboratory environment) positively selects for cells in an *agn43* ON state, and it has been hypothesized that during host colonization the same selective pressure for *agn43* ON cells will also exist (Chauhan *et al.* 2013). Antigen 43 has been shown to be functional when expressed in other bacterial species, in particular in *Pseudomonas fluorescens*, where it induces cell aggregation and biofilm formation (Kjaergaard *et al.* 2000a,b). It was also shown that when *E. coli* and *P. fluorescens* both express Ag43 they are able to co-aggregate and effectively form mixed species biofilms due to the interaction between Ag43 expressed on the cell surfaces of the two different species (Kjaergaard *et al.* 2000a,b).

The subfamily of autotransporter adhesins to which Ag43 belongs also contains two other *E. coli* produced adhesins that can enhance biofilm formation. The first, the autotransporter AIDA-I (AIDA for short) is an adhesin that is produced by some diarrheagenic *E. coli* strains that in its glycosylated form can adhere to a variety of mammalian cells (Benz and Schmidt 2001). AIDA is known to enhance cell aggregation and biofilm formation by *E. coli*, in a glycosylation-independent manner, through intermicrobial AIDA–AIDA interactions (Sherlock *et al.* 2004). It has been proposed that it is interactions between amino acids with charged side chains that may be responsible for the AIDA–AIDA interactions (Sherlock *et al.* 2004). It has also been shown that AIDA can interact with Antigen 43, causing the formation of cell aggregates containing both AIDA-expressing cells and Ag43-expressing cells, and these interactions are also AIDA-glycosylation independent (Sherlock *et al.* 2004). Another member of this autotransporter adhesion subfamily is TibA, which is produced by some strains of enterotoxigenic *E. coli* strains. Like AIDA, TibA is a glycoprotein, and glycosylation is essential for adherence of the *E. coli* strains to human cells, but similarly to the AIDA adhesin, glycosylation of TibA is not required for the aggregation of *E. coli* cells, or the enhancement of biofilm formation, caused by intermicrobial TibA–TibA interactions (Sherlock, Vejborg and Klemm 2005). Knowledge of the molecular basis of these interactions will be important in the development of small molecules with the potential to block cell-to-cell interactions during the infection process.

## CURLI—THE MAJOR PROTEIN COMPONENT OF THE BIOFILM MATRIX

**Figure 1.**
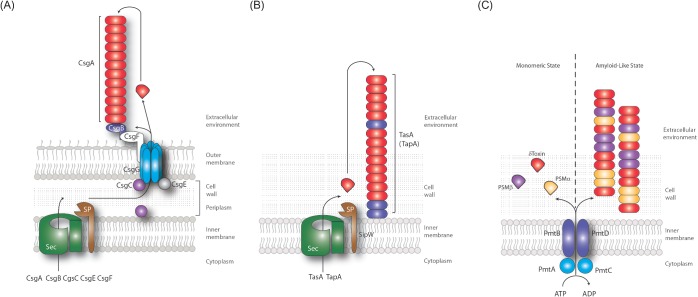
β-sheet-rich fibre formation by *E. coli, B. subtilis* and *S. aureus.* (**A**) Curli are amyloidous protein fibres assembled on the surface of *E. coli* cells within the nutrient-depleted zones of a biofilm and provide structural integrity. The curli fibre subunit CsgA is exported across the outer membrane through the CsgG translocator channel. Once outside the cell, CsgA interacts with the CsgB nucleator protein, and polymerizes into amyloidous fibres that extend away from the cell. The accessory proteins CsgC and CsgE regulate export by CsgG and CsgF is required for nucleation of CsgA by CsgB. **(B)** TasA-amyloid-like β-sheet-rich fibres protrude from the cell wall and are required for biofilm formation. Biogenesis requires the products of the *tapA-sipW-tasA* operon. Each protein is made in the cytoplasm and transported across the membrane by the Sec export system. SipW functions as a dedicated signal peptidase to cleave the signal peptide from TasA and TapA. TapA is required for anchoring the fibres to the cell wall and forms a minor component in the β-sheet-rich fibres. TasA is the major component in the fibres. **(C)** PSM β-rich fibres are found elaborated on the surface of *S. aureus*. The PSM transporter (PMT) is an ATP-dependent ABC transporter. It is composed of two transmembrane proteins (PmtB and D), coupled with the ATPases PmtA and PmtC. PSMs are known to function in the monomeric state where their surfactant activity has cytolytic activity. The formation of the fibre form is proposed to be a mechanism to inactivate the monomers until they are required again.

The major protein constituents of the *E. coli* biofilm matrix are the curli fibres. *Escherichia coli* thrives *ex vivo* and consistent with curli-dependent biofilm formation occurring outside the host, many isolates produce curli at temperatures below 30°C (Romling *et al.* 1998a). Curli are encoded for by two divergent operons, *csgBAC* and *csgDEFG* (Hammar *et al.* 1995) (Fig. 1A). The *csgBAC* operon encodes both components of the structural fibre, CsgA and CsgB, and the accessory periplasmic protein CsgC. The *csgDEFG* operon encodes the regulator CsgD, which controls curli and cellulose production, two putative accessory proteins CsgE and CsgF, and the translocator protein CsgG.

Curli fibres are composed of two proteins: CsgB functions to ‘nucleate’ polymerization of the fibre and makes up a minority component, whereas the majority of the fibre consists of CsgA (Hammar *et al.* 1995; Hammar, Bian and Normark 1996; Bian and Normark 1997) (Fig. 1A) (Table 1). The resulting curli fibres are rich in β-sheet structure and may be classified as ‘amyloid-like’, although alternative structures such as the β-helix are also consistent with the structural data (Shewmaker *et al.* 2009). Fibres bind the dyes Congo Red and Thioflavin T (ThT), which interact predominantly but not exclusively with β-sheet rich structures and aggregates (Khurana *et al.* 2001; Eisert, Felau and Brown 2006). The polymerized, fibrous form is highly resistant to denaturation and detergent solubilization (Collinson *et al.* 1991; Hammar, Bian and Normark 1996; Chapman *et al.* 2002).

**Table 1.** Functional amyloid/amyloid-like proteins in the biofilm matrix.

Bacterial species	Name of protein	Experimental evidence of amyloid properties	Function within biofilm	References
*Bacillus subtilis*	TasA (TapA minor component)	Electron and atomic force microscopy; Thioflavin T and Congo red binding propensity; CD spectrum profile	Biofilm matrix component	Romero *et al.* (2010); Chai *et al.* (2012)
*Enterobacter cloacae*	Curli (CsgA)	Similarity at gene level to *csg* operon; electron microscopy of whole cells	Biofilm matrix component	Zogaj *et al.* (2003)
*Escherichia coli*	Curli (CsgA)	Electron microscopy; Thioflavin T and congo red binding propensity; CD spectrum profile; NMR	Biofilm matrix component; adhesion	Chapman *et al.* (2002); Shewmaker *et al.* (2009)
*Pseudomonas* spp.	**F**unctional **a**myloid **p**seudomonas (Fap) fimbriae (FapC with FapB as a minor element)	Electron microscopy; CD spectrum profile; highly stable protein fibres	Biofilm matrix component	Dueholm *et al.* (2010, 2013)
*Salmonella* ssp.	Curli (alternatively Tafi for thin aggregative fimbrae) (CsgA)	Electron microscopy; highly stable protein fibres	Biofilm matrix component; resistance to antibacterial agents; adhesion to surfaces	Collinson *et al.* (1991); Collinson *et al.* (1996)
*Staphylococcus aureus*	Phenol Soluble Modulins	Electron microscopy; Thioflavin T binding propensity; detergent resistant fibre	Biofilm matrix component; biofilm stability; amyloid formation blocks dispersal activity of monomeric PSM *in vitro* under specific growth conditions; Not identified to date if synthesized *in vivo*	Periasamy *et al.* (2012); Schwartz *et al.* (2012)
*Streptococcus mutans*	Cell surface localized antigen P1 (PAc)	Electron microscopy; Thioflavin T and congo red binding propensity; detergent-resistant protein fibres	Biofilm matrix component	Oli *et al.* (2012)

The CsgA protein sequence comprises three regions: the Sec-signal peptide; an N-terminal 22 amino acid region of the mature protein that does not form an integral part of the fibre, but is required for stability and secretion (Collinson *et al.* 1999; Robinson *et al.* 2006; Nenninger *et al.* 2011); and the C-terminal domain that forms the core of the β-sheet-rich fibre (Collinson *et al.* 1999). This latter domain comprises five repeating units, predicted to encode the β-strands. The first and fifth of the repeating units of CsgA are essential for CsgA fibre-forming activity: when the first (N-terminal) repeat is deleted, CsgA shows very little fibre formation *in vitro*, whilst deletion of the fifth (C-terminal) repeat abolishes *in vitro* fibre formation completely (Wang, Hammer and Chapman 2008). *In vitro*, freshly purified His-CsgA is unstructured and unpolymerized, whereas curli fibres purified from *E. coli* are rich in β-sheet (Chapman *et al.* 2002).

CsgB has a similar overall domain structure to that of CsgA but the fifth, C-terminal repeat is less conserved than the other four, and additionally has a high proportion of positively charged amino acids. Deletion of either the fourth or fifth repeat resulted in a loss of outer membrane association and a corresponding loss of CsgA polymerization (Hammer, Schmidt and Chapman 2007). Nonetheless, the truncated version of CsgB, missing just the fifth repeating unit, was able to initiate CsgA polymerization *in vitro*, indicating that only the first four repeating units are required for its nucleation activity (Hammer, Schmidt and Chapman 2007). Like CsgA, CsgB in its monomeric form is unstructured, and only in an oligomerized state does it adopt a β-sheet rich secondary structure and enhance CsgA polymerization efficiency (Hammer, Schmidt and Chapman 2007). CsgB is associated with the outer membrane of *E. coli*, and in the absence of CsgA, forms short fibres on the surface of the *E. coli* cell (Bian and Normark 1997).

*In vitro* studies of the aggregation kinetics of CsgA, CsgB and mixtures of CsgA and CsgB revealed distinct kinetics for each curli component (Shu *et al.* 2012). In isolation, CsgB rapidly switches from unstructured to β-sheet, coincident with the onset of fibre formation. In comparison, CsgA initially forms amorphous aggregates with very little β-sheet structure, with fibres appearing only at later times. When CsgA and CsgB are mixed *in vitro*, CsgB rapidly self-assembles into oligomers that accelerate CsgA aggregation and fibre formation. This same process is thought to occur on the bacterial cell surface. Thus, current models of *E. coli* have CsgB anchored to the cell surface, where it nucleates the formation of CsgA fibres. Interestingly, CsgB is able to nucleate the formation of curli fibres when the CsgA protein is added exogenously (Wang, Hammer and Chapman 2008) or produced by other cells in the colony (Chapman *et al.* 2002). Thus, coproduction of CsgA and CsgB in the same cell is not required.

CsgG forms a pore within the outer membrane of the *E. coli* cell (Robinson *et al.* 2006), which is required for the secretion of CsgA, CsgB and CsgF (Loferer, Hammar and Normark 1997; Robinson *et al.* 2006; Nenninger, Robinson and Hultgren 2009; Taylor *et al.* 2011). CsgG contains a core domain attached to two adjacent transmembrane β-hairpins (Goyal *et al.* 2014). When extracted from the membrane using detergents, CsgG assembled into a nonamer forming a 40Å channel (Goyal *et al.* 2014), consistent with an earlier structural modelling approach that had suggested a channel formed of between 7 and 9 proteins (Taylor *et al.* 2011). A second structural analysis of CsgG once more revealed the CsgG channels to be composed of nine monomers, each of which contributed 4 β-strands to the final 36-strand β-barrel making up the pore through which the curli subunits are exported (Cao *et al.* 2014). Three aromatic residues (Phe-63, Tyr-66 and Tyr-71) were also identified as being important for the selectivity of the CsgG channel for the export CsgA and CsgB, and therefore are important for the assembly of functional curli (Cao *et al.* 2014). In the presence of all the protein components required for the formation of functional curli, CsgG forms foci in cells that colocalize with the point at which the curli fibres are anchored to the outer membrane (Epstein, Reizian and Chapman 2009). In the absence of any of the other components of the *csg* system, or with non-polymerizing mutants of CsgA, CsgG does not form foci and is instead dispersed around the cell (Epstein, Reizian and Chapman 2009). Overexpression of CsgG results in erythromycin sensitivity of the cells, but not vancomycin sensitivity or growth defects in the absence of antibiotics, revealing that overexpressed CsgG forms discrete pores in the membrane and does not cause a general loss of membrane integrity (Robinson *et al.* 2006).

The periplasmic protein CsgC is not required for curli assembly; however, *csgC* mutant strains exhibit defects in binding of Congo Red, loss of sedimentation (but not of aggregation) in static cultures and exhibit reduced binding to fibronectin (Hammar *et al.* 1995). Studies of the homologous *Salmonella* curli system Agf indicated that deletion of AgfC resulted in a different tertiary structure of the main curli subunit protein AgfA, and an absence of the nucleator protein AgfB (White *et al.* 2001; Gibson *et al.* 2007) (Table 1). The crystal structure of CsgC revealed an immunoglobulin-like fold with an invariant CxC motif in one of the loops between two of the β-strands (Taylor *et al.* 2011). This CxC motif placement was identified in another *E. coli* protein, DsbD, a periplasmic redox-active protein. This led Taylor *et al.* (2011) to hypothesize that CsgC may be involved in regulating the redox state of C230 of CsgG, thereby regulating the export of the fibre subunits and thus the formation of curli fibres.

CsgE is a periplasmic protein that functionally interacts with CsgG to facilitate curli fibre formation. In the absence of CsgE, CsgG forms an ungated pore through which erythromycin enters the cell and non-curli proteins are secreted (Nenninger *et al.* 2011). This suggests that CsgE provides a gating mechanism to prevent uncontrolled protein secretion and/or small molecule uptake via the CsgG pore. The first 22 amino acids of CsgA, previously shown to be sufficient to direct CsgA to the CsgG translocon (Robinson *et al.* 2006), act as a specificity signal to allow translocation through the CsgG pore when gated by CsgE (Nenninger *et al.* 2011).

CsgE has additionally been shown to prevent the self-assembly of CsgA into fibres *in vitro* (Nenninger *et al.* 2011), thereby acting as a chaperone to prevent inappropriate self-assembly. When added to CsgA *in vitro* at any point during fibre formation, CsgE prevents further fibre formation or growth, and surface plasmon resonance experiments indicated that this is due to a direct interaction between CsgE and the CsgA fibres (Andersson *et al.* 2013). Moreover, exogenously added CsgE inhibited the formation of floating, pellicle, biofilms, a process that is dependent on the formation of curli fibres (Cegelski *et al.* 2009; Andersson *et al.* 2013).

CsgF is required for nucleation of CsgA by CsgB, and for the formation of curli fibres (Chapman *et al.* 2002). CsgF localizes to the outer surface of the outer membrane, where it directly interacts with the translocator protein CsgG (Robinson *et al.* 2006; Nenninger, Robinson and Hultgren 2009). Colonies formed by a *csgF*^−^ strain do not stain with Congo Red, and fewer fibres are observed. The majority of the CsgA protein remains in an SDS-soluble, unaggregated, form (Chapman *et al.* 2002) that is not cell associated (Nenninger, Robinson and Hultgren 2009). In a *csgG*^−^ strain, CsgF is undetectable from whole-cell samples, but neither of the curli fibre subunits CsgA and CsgB nor the accessory protein CsgE was required for CsgF localization at the cell surface (Nenninger, Robinson and Hultgren 2009). CsgF enhances the cell-surface localization of CsgB and has also been shown to mediate the protease resistance of CsgB, which in turn is associated with the ability of CsgB to nucleate CsgA (Nenninger, Robinson and Hultgren 2009). CsgF, like CsgA and CsgB, contains a high proportion of glutamine and asparagine residues, and it has been hypothesized that these residues promote CsgB structure formation, although this remains to be experimentally tested (Nenninger, Robinson and Hultgren 2009).

Whilst the majority of the molecular studies on curli biogenesis and function have focused on those from *E. coli* (and *Salmonella*), a bioinformatics-based approach has identified curli homologs throughout the Proteobacteria, as well as in some Bacteroidetes, a single Firmicutes species, Halanaerobium and a member of the Thermodesulfobacteria (Dueholm *et al.* 2012). In addition to identifying homologs of the curli structural proteins CsgA and CsgB, Dueholm *et al.* (2012) also analysed the conservation of the *csgCAB* and *csgDEFG* operon structures, showing that whilst *csgEFG* were found in most of the bacteria with potential CsgA/B homologs, the accessory protein CsgC and the regulator CsgD were often missing. Whilst this bioinformatics approach remains to be validated by *in vivo* analysis of the identified curli homologs in other species, it does suggest that the formation of curli-like fibres may be more widespread throughout biofilm-forming bacteria than first thought, forming a common mechanism to provide structural integrity to the biofilm matrix. Analogous extracellular protein fibres involved in biofilm formation are detailed in Table 1.

## CELLULOSE

Cellulose is one of the most abundant organic polymers in nature (Ljungdahl and Eriksson 1985). It has a relatively simple structure, made from a (1 → 4)-β-linked linear glucose chain and, while most frequently thought of in connection with the plant cell wall, it can be synthesized by some species of bacteria where it has a protective, architectural and regulatory function during biofilm formation (Solano *et al.* 2002; Ude *et al.* 2006; Gualdi *et al.* 2008). The role of cellulose as a component of the biofilm matrix was first identified in *Salmonella typhimurium*, in which the cellulose synthase gene cluster which is conserved between *S. typhimurium* and *E. coli* was initially identified (Zogaj *et al.* 2001). In the same study, homologues of the cellulose gene cluster were noted in four different *E. coli* strains, three of which were demonstrated to produce cellulose (Zogaj *et al.* 2001). Further analysis of cellulose production by *E. coli* revealed that the proteins involved in cellulose synthesis are encoded in two divergent operons: *yhjR*-*bcsQABZC* and *bcsEFG* (Zogaj *et al.* 2001; Solano *et al.* 2002; Le Quere and Ghigo 2009). The BcsA and BcsB proteins form the two subunits of the cellulose synthase complex that is located within the membrane and converts UDP-glucose to cellulose (Zogaj *et al.* 2001; Omadjela *et al.* 2013).

While cellulose is clearly needed for biofilm formation (as discussed further below), in the *E. coli* strain MG1655, overproduction of cellulose has a negative effect on submerged biofilm formation (in 96-well plates and glass tubes) and on cell aggregation (Gualdi *et al.* 2008). It was proposed that, when overexpressed, cellulose may inhibit the formation of biofilm types that are reliant on curli-mediated adhesion by masking the curli fibres by coating them in excessive cellulose (Gualdi *et al.* 2008). Here, it was also revealed that overexpression of cellulose increased resistance of *E. coli* to desiccation, whilst deletion of the gene encoding the major curli subunit CsgA resulted in a decrease in desiccation resistance. It was proposed that curli and cellulose may work together to protect the *E. coli* against environmental stresses (Gualdi *et al.* 2008). A recent study has shown that biofilm formation by *E. coli* protects the bacteria from predation by both the nematode *Caenorhabditis elegans* and by the predatory bacterium *Myxococcus xanthus*, and that this protection is dependent on the presence of both cellulose and curli (DePas *et al.* 2014).

## A STRUCTURAL ROLE FOR THE FLAGELLUM WITHIN THE DEVELOPING *E. COLI* BIOFILM

**Figure 2.**
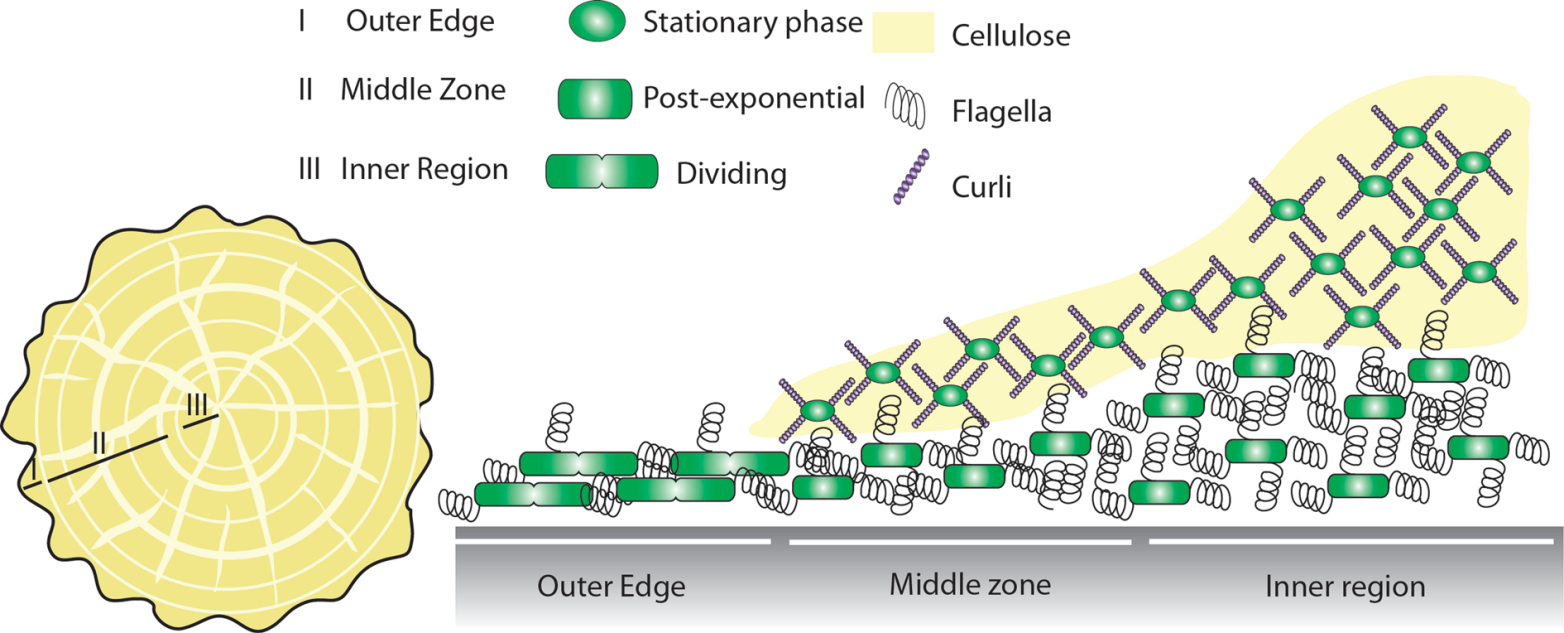
*Escherichia coli* biofilm structure. After prolonged incubation, *E. coli* forms complex colony type biofilms on agar plates, where the three major components of the biofilm matrix have been shown to be curli fibres, cellulose and flagella filaments. Left: the *E. coli* complex colony contains both concentric rings and axial wrinkles, the rings are dependent on the production of both curli and flagella and the axial wrinkles additionally require the production of cellulose. The colony can be divided into three zones (I) the outer edge, (II) the middle zone and (III) the inner region. Right: a cross-section of the colony shows the different cell types co-existing within the biofilm, and their location within the different biofilm regions. Dividing, flagellated cells are found within the outer edge, whilst in the middle zone and the inner region two distinct cell types are found. Near the agar surface post-exponential, rod-shaped cells are found encased in a mesh of flagella filaments, whilst in the upper levels of the colony stationary phase, ovoid cells are found, these are surrounded in a dense mesh of curli fibres and cellulose.

*Escherichia coli* is capable of forming architecturally complex macrocolonies on agar plates (Romling *et al.* 1998b; Serra *et al.* 2013b) that are structurally reminiscent of the biofilms formed by, for example, both *B. subtilis* (Branda *et al.* 2001) and *Vibrio cholerae* (Yildiz and Schoolnik 1999). The molecular mechanisms underpinning the macrocolony formation process have been probed using genetically distinct strains of *E. coli* and architectural complexity has been proven to be provided by three components: the curli fibres and cellulose as described above, but also the bacterial flagellum (Hung *et al.* 2013; Serra, Richter and Hengge 2013a; Serra *et al.* 2013b). It was during analysis of the macrocolony structures formed by the *E. coli* K12 strain W3110 that the structural role for the flagellum during biofilm formation was revealed. It is important to note that in strain W3110, as with all widely used laboratory isolates of *E. coli*, cellulose is not produced due to a point mutation in the *bcsQ* gene (Serra, Richter and Hengge 2013a). The macrocolony formed by strain W3110 can be divided into three distinct regions: the outer edge, which forms a narrow and smooth zone; a middle zone in which wrinkles initially emerge and which then becomes characterized by the appearance of concentric rings; and the inner wrinkled region, corresponding to the area in which cells were first inoculated onto the plate (Fig. 2). Experimental work indicated that the morphology of the developing biofilm was dependent on both the production of the flagella filament and on the ability of the flagella to rotate. Mutation of either *fliC* or *motA* (the flagellar filament protein and one of the motor proteins) rendered *E. coli* unable to make a fully ringed patterned colony (Serra *et al.* 2013b). In contrast, analysis of mutants in the regulatory cascade that govern flagella synthesis, *flhDC* or *fliA*, (which do not produce flagella) resulted in macrocolonies with the ring structures but which lacked wrinkles (Serra *et al.* 2013b). In the *flhDC* and *fliA* deletion strains, there is an increase in the level of the secondary messenger cyclic-di-GMP due to the loss of expression of phosphodiesterase YhjH which is under the control of both FlhDC and FliA (Pesavento *et al.* 2008; Serra *et al.* 2013b). The increase in cyclic-di-GMP levels results in increased curli production which partially compensates for the absence of the flagellar filament, presumably by fulfilling its structural role (Pesavento *et al.* 2008; Serra *et al.* 2013b). Microscopy analysis of the internal structure of the colony biofilm revealed that cells in the lower portion of the biofilm, nearest the agar, were surrounded in a network of flagella filaments, where it is hypothesized that the flagella filaments are acting as a structural component of the biofilm matrix (Serra *et al.* 2013b). In a *motA* mutant strain, where the flagella are unable to rotate, the mesh of flagella filaments were seen to be less entangled, suggesting that flagella rotation is required for effective tethering together of the cells and enables the formation of the mesh of flagella filaments at the base of the macrocolony (Serra *et al.* 2013b). These findings link the flagellar filaments with the overall architecture of the biofilm and reinforce the regulatory role for flagellar biosynthesis in the coordination of transcription of the other matrix elements.

Analysis of floating, pellicle, biofilms formed at the air–liquid interface revealed a further requirement for flagella in biofilm formation (Hung *et al.* 2013). Strains harbouring deletions of either the gene encoding the filament subunit FliC or the master regulator FlhDC were severely attenuated in their ability to form pellicle biofilms (Hung *et al.* 2013). Both strains formed small ‘rosette’ biofilms at the air–liquid interface. Analysis of the protein content of the wild-type pellicle biofilms revealed a complete absence of FliC protein, suggesting that flagella are not used as a structural component in the pellicle form of biofilm, in contrast to in the macrocolony (Hung *et al.* 2013; Serra *et al.* 2013b). This suggests that the main role of flagella in pellicle formation is in the initial aggregation of the cells at the air–liquid interface. It is likely that in the natural habitat the requirement for the flagellum is variable and will encompass physical propulsion, regulatory and architectural roles.

## *E. COLI* PRODUCES STRUCTURAL COMPONENTS OF THE MATRIX IN DISCRETE ZONES OF THE BIOFILM

Insight to the spatial distribution of cells in the *E. coli* macrocolony came from a combination of SEM and fluorescence microscopy of cross-sections (Serra, Richter and Hengge 2013a; Serra *et al.* 2013b). The images demonstrated that curli fibres and flagella are synthesized in discrete zones of the biofilm, highlighting a bimodality that developed within an isogenic progenitor population (Serra *et al.* 2013b) (Fig. 2). At the upper surface of the biofilm, the cells were small, ovoid and surrounded in an extracellular mesh of curli fibres (Serra *et al.* 2013b). By comparison, the cells at the bottom of the macrocolony, nearer to the surface of the agar plate, were longer and rod shaped. Again these cells were encased in a dense mesh of filaments; however, in this case the mesh was composed of flagella filaments (Serra *et al.* 2013b). Zoning of cell types was also apparent horizontally through the biofilm macrocolony (Fig. 2). The outer edge zone of the macrocolony contained long rod-shaped cells that were again tethered together in a mesh of entangled flagella. Within the middle zone of the macrocolony (the area in which the concentric rings form), an interface was seen where cells encased in the mesh of flagella at the base of the colony gave way to smaller ovoid cells surrounded in a dense mat of curli fibres in the upper regions. The composition of the matrix mesh seems to correlate with the metabolic state of the cells within that region of the biofilm. In the outer edge zone of the macrocolony, and in the lower areas nearer to the agar surface, the rod-shaped cells are in a state of post-exponential growth and are still dividing, and are also still able to produce flagella. In the upper, central zones of the biofilms the cells have entered stationary phase due to nutrient limitation, causing them to stop producing flagella, and instead become smaller ovoid cells that produce curli (Serra *et al.* 2013b).

Restoration of cellulose production to the laboratory strain of *E. coli* K12 W3110 changed not only the morphology of the colony, but also its size and overall physical properties. The colony formed by the dual curli- and cellulose-producing isolate was flat, large and showed a pronounced ‘tissue-like’ elasticity (Serra, Richter and Hengge 2013a). In the upper zones of the biofilm, in the areas previously shown to consist of small ovoid cells encased in a curli-fibre mesh, the addition of cellulose production resulted in the smooth coating of all the cells in this region, suggesting that both cellulose and curli contribute to the matrix structure. In the transition zone, between the stationary phase, curli-producing cells in the upper layer and the post-exponential, flagella-producing cells in the lower layer, a zone rich in cellulose was observed. In this transition zone, the entangled flagella were observed to be acting as a scaffold for cellulose fibre formation (Serra, Richter and Hengge 2013a). It is relevant to note that cellulose can ‘wheel lock’ flagellar rotation, thereby leading to an alteration in gene regulation that turns ‘off’ flagellar gene transcription (Zorraquino *et al.* 2013). This physically mediated method of impeding biogenesis of the flagellum allows production of cellulose to be tightly linked with the onset of motility inhibition.

The demarcation of different cell types in a stratified *E. coli* biofilm has also been seen for the uropathogenic *E. coli* strain UTI89 which forms floating pellicles at the air–liquid interface. Using a combination of scanning and transmission electron microscopy, two distinct zones were identified within the pellicle biofilm, the upper air-exposed side and the lower liquid-exposed side (Hung *et al.* 2013). These two zones are reminiscent of those identified in the macrocolony formed by *E. coli* K12 (Serra, Richter and Hengge 2013a). At the upper, air-exposed, side of the pellicle, the (apparently ovoid) cells were covered in a matrix composed of very densely packed fibres that formed ‘basket-like’ structures surrounding the *E. coli* in the biofilm (McCrate *et al.* 2013; Serra, Richter and Hengge 2013a). At the base of the pellicle, nearest to the liquid, the cells were seen to be rod shaped and loosely packed, with very little fibrous matrix around the cells. As indicated previously, flagella were not detectable by Western Blot analysis of the pellicular material, so all the fibrous material within the matrix is likely to be formed from a combination of curli and cellulose (Hung *et al.* 2013). The insoluble extracellular matrix material extracted from these biofilms has been subjected to state-of-the-art solid-state nuclear magnetic resonance (NMR) analysis, and the ratio (by mass) of the curli fibres to cellulose was shown to be consistently approximately 85%:15% (McCrate *et al.* 2013).

Further analysis of the localization of matrix components, and of the metabolic states of cells within the biofilm regions, of the biofilms formed by other related Enterobacteriaceae will reveal whether the mechanisms employed by *E. coli* in biofilm formation are universally adopted strategies and will highlight the *in vivo* relevance. Whilst the distinct cell types within the biofilms are likely caused by the differences in nutrient availability across the biofilm, i.e. the post-exponential-phase cells located nearer the nutrient source, and the stationary-phase, curli and cellulose producing, cells further away from the nutrients, this diversification of cell fate may act as a protective or bet-hedging solution allowing for a more rapid response to environmental changes or stresses as there already exist cells in a range of metabolic states.

### VIBRIO CHOLERAE

*Vibrio cholerae* is a Gram-negative halophilic bacterium with a single polar flagellum that is a member of the family Vibrionaceae. It is the etiological agent of cholera, a disease which results in severe, and often fatal, diarrhoea. The formation of biofilm communities is important in the disease process from both the angle of initial infection and transmission. The main route for infection is the consumption of contaminated food or water (Zuckerman, Rombo and Fisch 2007). It has been shown that removal of particulate matter from water sources using crude filtration methods that remove aggregates of bacteria or biofilms significantly reduces the prevalence of cholera infection (Huq *et al.* 1996; Colwell *et al.* 2003). Consistent with these findings, it has been shown that biofilm-like masses extracted from the faeces of infected patients are more infectious than planktonic bacterial counterparts (Faruque *et al.* 2006; Kamruzzaman *et al.* 2010). Here we will discuss recent advances in the understanding of the structure and function of the extracellular polysaccharide (EPS) and protein components of the *V. cholerae* biofilm matrix and highlight major novel experimental approaches that have altered our perspective of how the biofilm matrix is assembled.

## COMPOSITION OF THE *VIBRIO* EXOPOLYSACCHARIDE

The toolbox of molecular components used by *V. cholerae* to form the biofilm matrix has been identified predominantly using classical bacterial genetic approaches (Yildiz and Schoolnik 1999). The major biofilm exopolysaccharide, dubbed VPS for *Vibrio* exo*p*oly*s*accharide, was first recognized during molecular analysis of a rugose colonial variant of *V. cholerae* O1, biotype El Tor (Yildiz and Schoolnik 1999). It was established that the rugosity of colony and pellicle morphology was directly correlated with enhanced production of an EPS and that this additionally increased resistance to chlorine, suggesting a protective function for the exopolymer (Yildiz and Schoolnik 1999). Since this initial discovery, it has been demonstrated in various studies that VPS is required for biofilm formation under many *in vitro* conditions using pellicle and colony formation assays as indicators (Fong *et al.* 2010). Moreover, in support of biofilm formation and VPS production being required for infection and biofilm formation *in vivo*, analysis using the rabbit ileal loop model system demonstrated poor biofilm formation by the *vps* mutant (Kamruzzaman *et al.* 2010). Further to this, colonization of the intestine of *Drosophila melanogaster* after oral ingestion of *V. cholerae* occurs in a VPS-dependent manner, although the physiological relevance remains to be determined given that *Drosophila* is not a natural vector for transmission (Purdy and Watnick 2011). It is important to note, however, that *V. cholerae* has the capacity to form two types of biofilms in the laboratory; one that is dependent on VPS and another that is VPS independent and triggered by concentrations of Ca^2+^ found in seawater (Yildiz and Schoolnik 1999; Kierek and Watnick 2003a,b). These findings are compatible with a scenario where in the natural environment the VPS is required for biofilm formation in some environmental conditions while not in others and is likely compensated for by the proteinaceous components that are discussed below.

Building on the identification of VPS as an important component of the biofilm matrix, a necessary prelude to chemical disruption of biofilms using small molecules to disrupt or digest the VPS is detailed knowledge of the structure. The genes that participate in the biosynthesis of the *V. cholerae* exopolysaccharide are found in two clusters on the chromosome (Fong *et al.* 2010). Cluster *vps*-I consists of the genes *vpsU* (VC0916) and *vpsA-K* (VC0917–27) and cluster *vps*-II carries the *vpsL-Q* (VC0934–9). Using a systematic mutagenesis approach, it was identified that the single mutant strains fell into six phenotypic classes with the majority of the 18 genes in the two clusters being involved or required for VPS production, and therefore biofilm formation (Fong *et al.* 2010). Initial compositional examination of isolated VPS suggested an approximately equal contribution from glucose and galactose monomers, with linkage analysis detecting a similar amount of 4-linked galactose and 4-linked glucose, suggesting that they formed the backbone of the molecule with branching of 3,4- and 4,6-linked galactose and glucose and 2,4-linked galactose forming the side chains (Yildiz and Schoolnik 1999). However, solution-state NMR analysis has recently elucidated the structure of the polysaccharide component of the VPS and in doing so established the presence of an additional sugar monosaccharide (namely 2-acetamido-2-deoxy-L-guluronic acid) in the polysaccharide (Yildiz *et al.* 2014). These findings expand the chemical space of molecules used in *Vibrio* biofilm biology and demonstrate the complexity of understanding the nature of the exopolysaccharides found in the matrix. Additional analysis indicated that the VPS isolated from the biofilm matrix appears bound to an additional as yet unidentified component that increases the solution viscosity and thereby suppresses any solution NMR signal (Yildiz *et al.* 2014). After acid hydrolysis, the polysaccharide component released was amenable to NMR spectroscopy. It will be of interest to define the interaction between the VPS and the unknown molecule and establish if this interaction plays a critical role in biofilm matrix assembly and integrity. Moreover, it is clear that further work will be required before targeted enzymatic digestion of the VPS in biofilm aggregates can be used as a therapeutic approach.

The study of VPS in *Vibrio* highlights the strength of new technology. For example, an advance in the understanding of the molecular composition of the *V. cholerae* biofilm matrix has been achieved using novel solid-state NMR methodologies (Reichhardt *et al.* 2014). This new approach avoids the necessity to treat samples with harsh chemicals that are needed for other forms of analysis such as MS/MS analysis of carbohydrate composition. The new atomic-level information of the biofilm matrix of *V. cholerae* has provided a molecular fingerprint in a largely unprocessed state allowing the assignment of the carbon pool to sugar, lipid and protein pools thereby conclusively demonstrating that, unlike the rugose *E. coli* biofilm (McCrate *et al.* 2013), the majority of the biofilm matrix is in carbohydrate form (Reichhardt *et al.* 2014). The development of this technique adds to the pool of methodologies that allow an analysis of the macromolecules in the extracellular matrix *in situ* (Nichols *et al.* 1985; Ivleva *et al.* 2008, 2010; Lanni *et al.* 2014; Neu and Lawrence 2014). It is of interest to note that these methods of analysis are entirely compatible with molecular investigations of the impact of specific genetic mutations, the analysis of mixed species and complex natural communities and additionally could shed light on the molecular composition of the biofilm matrix formed by genetically intractable species.

## PROTEIN COMPONENTS IN THE *V. CHOLERAE* BIOFILM MATRIX

The biofilm matrix of *V. cholerae*, like the majority of studied bacterial species, also contains extracellular proteins that contribute to biofilm structure and integrity. There are three main proteins involved in the *V. cholerae* biofilm matrix structure and integrity namely Bap1, RbmA and RbmC. Each of these proteins is synthesized with an N-terminal signal peptide and is secreted into the biofilm matrix by the type-II secretion system (Johnson *et al.* 2014). Consistent with an extracellular localization, proteomic analysis of *V. cholerae* outer membrane vesicles (OMV) has reported that RbmA, RbmC and Bap1 are OMV proteins. Furthermore, incorporation of the proteins into the OMVs was shown to be dependent on the protease DegP (Altindis, Fu and Mekalanos 2014). Here we will discuss what is known about the structure and function of each of the aforementioned proteins.

**Figure 3.**
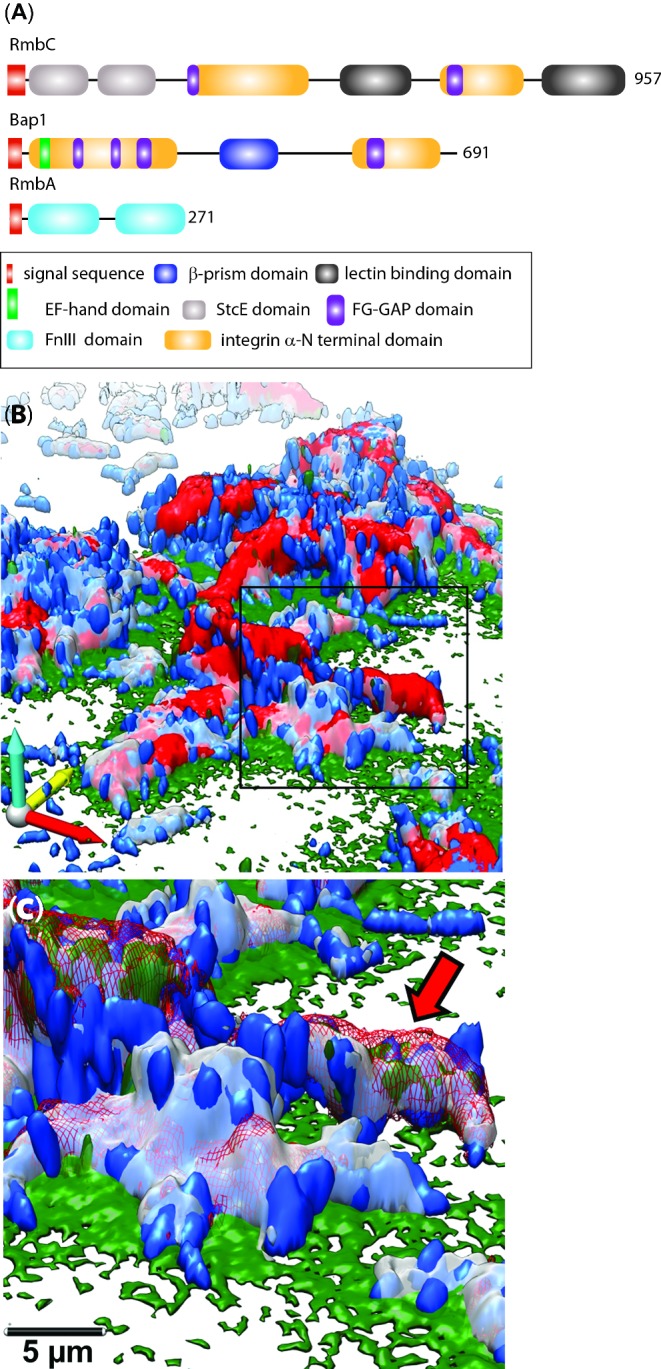
Structure and deployment of *V. cholerae* biofilm matrix proteins. (**A**) Bioinformatics analysis of RmbC (accession number Q9KTH2), RmbA (accession number Q9KTH4) and Bap1 (accession number Q9KQW0) was performed *de novo* using a combination of SMART (Schultz *et al.* 1998; Letunic, Doerks and Bork 2014), InterPro (Hunter *et al.* 2012) and BLAST (Johnson *et al.* 2008) to identify conserved domains, SignalP (Petersen *et al.* 2011) to designate signal sequence peptide cleavage sites and where required further information was revealed using WU-BLAST analysis (http://www.ebi.ac.uk/Tools/sss/wublast/). For Bap1, it should be noted that the EF-hand domain has a low confidence score of 7.00e-03 and for RmbC the integrin α-N-terminal domain had a confidence value of 5.00e-04 so the presence of these protein domains should be interpreted with caution. The domains and proteins are drawn approximately to scale. Parts **B** and **C** are reproduced, with permission, from Berk *et al.* (2012) Science along with the corresponding legend. Images are pseudo-coloured blue (cells), grey (RbmA), red (RbmC) and green (Bap1). RbmA localizes around and within cell clusters. RbmC and Bap1 encase cell clusters. Cells were counterstained with DAPI. Scale bars, 3 μm. (B) 3D biofilm architecture. (C) Enlargement of the boxed region in (B). Red arrow indicates one cell cluster. Red signal now rendered partially transparent to allow visualization of cells within an RbmC-containing cluster.

Bap1 (for *b*iofilm-*a*ssociated *p*rotein 1) was initially uncovered using a global transcriptomic analysis designed to identify genes that were differentially expressed between biofilm- and monolayer-attached cells (Moorthy and Watnick 2005). Bap1 is encoded by locus VC1888 and is coregulated with the *vps*-island (Moorthy and Watnick 2005). Bap1 has been linked with anti-microbial resistance in a process currently thought to be independent of its role in biofilm formation (Duperthuy *et al.* 2013). Bap1 is a large protein (691 amino acids) and detail regarding the mechanism of Bap1 is limited, and knowledge is largely restricted to that gleaned from bioinformatic analyses (Fig. 3A). This has identified the presence of multiple FG-GAP domains (Fong *et al.* 2006; Absalon, Van Dellen and Watnick 2011) and a β-prism domain in the sequence (Fig. 3A), but the function of these domains is currently unknown. Recent biophysical analysis has linked Bap1 with controlling the mechanical strength of pellicle biofilms and the overall hydrophobicity of the air–liquid interface structures. These data were generated using interfacial rheology to measure the elasticity of pellicle biofilms and contact angle measurements to assess the surface properties (Hollenbeck *et al.* 2014). These findings place Bap1 in the same functional category as proteins such as BslA of *B. subtilis* (*vide infra*) (Kobayashi and Iwano 2012; Hobley *et al.* 2013).

RbmC (for *r*ugosity and *b*iofilm structure *m*odulator *C*) is 957 amino acids in length and exhibits 54% identity and 70% similarity to Bap1 (Absalon, Van Dellen and Watnick 2011). Consistent with this high level of homology, Bap1 and RbmC are functionally redundant during biofilm formation. Like Bap1, RbmC also contains multiple FG-GAP domains and additionally harbours two lectin-binding domains, suggestive of carbohydrate-binding capability (Fig. 3A). Deletion of the C-terminal lectin-binding (β-prism-like) domain of RbmC, that is absent from Bap1, indicates that this domain is dispensable for function (Absalon, Van Dellen and Watnick 2011). These findings thereby support the data indicating that the two proteins can functionally compensate for each other but leave open the question of which regions of the protein are mechanistically important. Despite the apparent functional redundancy, high-resolution image analysis documenting the timing of protein production and localization in the biofilm matrix has highlighted differences in the deployment and localization of the two proteins (Berk *et al.* 2012).

RbmA (for *r*ugosity and *b*iofilm structure *m*odulator *A*) is needed for biofilm rugosity and detergent resistance (Fong *et al.* 2006). It is a 271-amino-acid protein that is synthesized with a 30-amino-acid Sec-dependent signal peptide (Fig. 3A) and is secreted into the extracellular environment via the type II secretion system (Johnson *et al.* 2014). RbmA was initially identified using proteomic analysis as increased in production within rugose biofilms (Fong *et al.* 2006) and consistent with a role in biofilm formation it is encoded in a region of the large chromosome along with the *vps*-gene clusters. Atomic-level information regarding RbmA function has been gleaned from the recently solved structure of the mature domain (Giglio *et al.* 2013). Here it was elucidated that RbmA forms a homodimer where each monomer comprises two tandem fibronectin type (FnIII) domains (Fig. 3A). Biological significance for the dimer observed in the crystal structure was determined biochemically using size exclusion chromatography and small angle X-ray scattering (Giglio *et al.* 2013). Analysis of the tertiary structure identified significant homology with several proteins including GpbA from *V. cholerae* which is an *N*-acetylglucosamine (GlcNAc)-binding protein, a human transglutaminase and a dextranase from *Streptococcus mutans* (Giglio *et al.* 2013). Based on the knowledge that some of the structurally related proteins are known to bind carbohydrates, structure-guided site-directed mutagenesis was used to isolate functionally active domains of the protein. The importance of a surface-exposed strong positive electrostatic groove for rugose biofilm formation was identified, and it was postulated that this region was required for interactions with other matrix components, such as the VPS, to facilitate the function of RbmA as a scaffold in the biofilm matrix (Giglio *et al.* 2013). This is a strong hypothesis that is consistent with the knowledge that protein–carbohydrate interactions are required for biofilm stability and formation. One example that falls in this class is CdrA which is an extracellular β-helical filamentous protein synthesized by *P. aeruginosa* that interacts with an exopolysaccharide called Psl to consolidate the matrix (Borlee *et al.* 2010). As a multivalent adhesion, CdrA has the potential to interact with more than one component presumably thereby strengthening the cross-links within the biofilm matrix and the range of interactions possible with a host.

## NOVEL *IN SITU* IMMUNOFLUORESCENCE ANALYSIS OF PROTEIN LOCALIZATION

Building on the knowledge of the proteins found in the *V. cholerae* biofilm matrix, exciting data showing where and when the proteins are localized within the architecture of the biofilm have recently been derived using immunofluorescence imaging technologies (Absalon, Van Dellen and Watnick 2011; Berk *et al.* 2012). Initial work using single colour immunofluorescence microscopy identified that RmbA was localized throughout the entire biofilm whereas Bap1 was restricted to the base of the biofilm where it mediates adhesion of neighbouring ‘bystander’ cells (Absalon, Van Dellen and Watnick 2011). Subsequently, greater resolution of the timing and position of deployment was achieved by employing powerful high-resolution four-colour immunofluorescence confocal microscopy. As with the single protein analysis method, this approach required the use of a genetically tractable organism. Modification of the coding regions for RmbA, RmbC and Bap1 was required so that each incorporated a unique immunoreactive epitope tag. The production of the epitope-tagged proteins was subsequently followed, in real time, during biofilm formation using multi-resolution confocal microscopy. This showed that each of the components in the biofilm matrix had complementary roles that, in combination, facilitated the assembly of the biofilm. The first protein that was detected was RmbA, which was found in discrete sites on the cell surface and allowed daughter cells to remain attached to the surface. Next was Bap1, which localized to the junction between two daughter cells promoting retention of the new cell and coated the surrounding surfaces. Finally, RmbC was detected at discrete sites on the cell surface which, over time, developed into a layer that encapsulated clusters of cells alongside the VPS and Bap1 (Berk *et al.* 2012) (Fig. 3B and C). It will be of interest to establish if similar patterns of protein behaviour are observed for other species allowing classification of the proteins in the biofilm matrix into functional subgroups in the matrix.

## BACILLUS SUBTILIS

**Figure 4.**
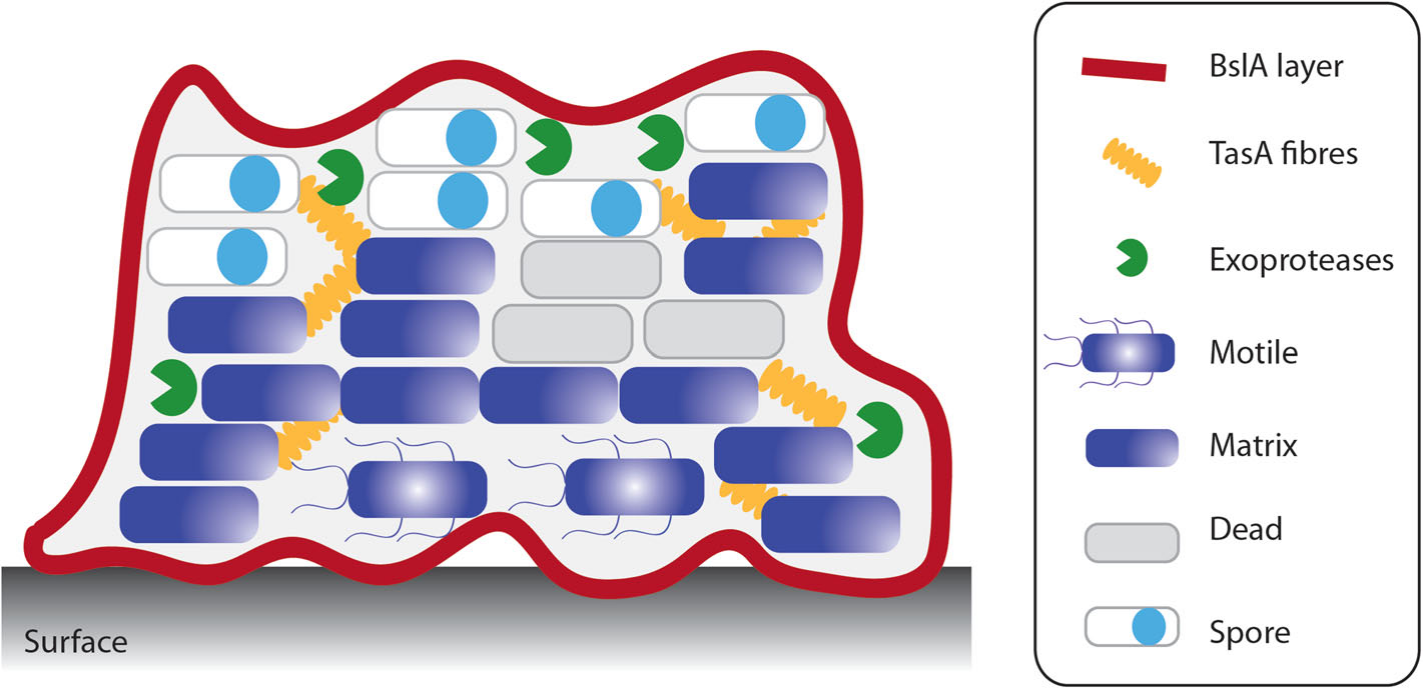
*Bacillus subtilis* biofilm formation. Biofilm formation by *B. subtilis* culminates in the formation of a structured highly hydrophobic sessile community. The isogenic population differentiates to divide tasks within the community. For a detailed review of this process refer to (Cairns, Hobley and Stanley-Wall 2014; Mielich-Suss and Lopez 2014).

*Bacillus subtilis* is a Gram-positive endospore forming soil bacterium that is used to study the molecular mechanisms of bacterial development. Commercially, *B. subtilis* and the closely related species *B. amyloliquefaciens* are known as plant growth-promoting bacteria that can protect plants from pathogenic microorganisms in a manner dependent on biofilm formation (Bais, Fall and Vivanco 2004; Kloepper, Ryu and Zhang 2004; Nagorska, Bikowski and Obuchowski 2007). It was initially reported that *B. subtilis* had the capacity to form what are now defined as ‘biofilms’ back in the 1870s by Burton-Sanderson and Ferdinand Cohn (Cohn 1877; Vlamakis *et al.* 2013); however, it was only in the early 2000s that investigations into the molecular basis of biofilm formation were initiated. The work started with the identification of the capability of environmental isolates of *B. subtilis* to form rugose colonies and pellicles that were subsequently shown to contain subpopulations of isogenic differentiated cells (Fig. 4A) (Branda *et al.* 2001; Vlamakis *et al.* 2008; Marlow *et al.* 2014). At the same time it was noted that laboratory isolates of *B. subtilis* had lost the capability to form rugose biofilms (Branda *et al.* 2001) but retained the capacity to develop submerged surface-adhered biofilms (Hamon and Lazazzera 2001). The survival and propagation of *B. subtilis* in the biofilm is dependent on the production of an extracellular matrix. The matrix is required for the projection of environmentally resistant spores on aerial structures at the surface of the biofilm (Branda *et al.* 2001; Veening, Hamoen and Kuipers 2005), wrinkle and internal channel formation (Wilking *et al.* 2013), complex colony spreading (Seminara *et al.* 2012), extreme hydrophobicity (Epstein *et al.* 2011) and mechanical stiffness (Asally *et al.* 2012).

The *B. subtilis* biofilm matrix predominantly comprises a large molecular weight soluble secreted polysaccharide, for which there is limited compositional knowledge (Branda *et al.* 2001; Chai *et al.* 2012; Jones *et al.* 2014), and extracellular proteins called TasA and TapA (Branda *et al.* 2006; Romero *et al.* 2011). The production of the aforementioned extracellular molecules is not sufficient to accomplish biofilm maturation, which does not occur unless the protein BslA (*B*iofilm **s**urface *l*ayer protein *A*) (originally called YuaB) is synthesized (Ostrowski *et al.* 2011). It is noteworthy that in line with biofilm matrix production being energetically expensive, the expression of each of the operons needed for matrix biosynthesis is subject to tight transcriptional control. The regulatory pathways that control transcription and the current knowledge of the EPS structure and function have recently been reviewed (Cairns, Hobley and Stanley-Wall 2014; Mhatre, Monterrosa and Kovacs 2014; Mielich-Suss and Lopez 2014). Thus, here we focus on the current understanding of the mechanisms underpinning the production and function of each of the protein matrix components.

## A NOVEL BACTERIAL HYDROPHOBIN IS USED TO MAKE A BIOFILM RAINCOAT

BslA is a bacterial hydrophobin that is needed for the observed architectural complexity of the rugose wild-type biofilm and for the formation of a highly hydrophobic barrier that encases the bacterial community (Kobayashi and Iwano 2012; Hobley *et al.* 2013). Consistent with this function, by using both immunofluorescence and epifluorescence microscopy, BslA was found to form a coat or layer around the mature biofilm (Kobayashi and Iwano 2012; Hobley *et al.* 2013). BslA production is highly regulated at the level of transcription (Kobayashi 2007; Verhamme, Murray and Stanley-Wall 2009; Kovacs and Kuipers 2011) and functions cooperatively with the TasA/TapA and exopolysaccharide components of the matrix to expedite biofilm maturation (Ostrowski *et al.* 2011; Kobayashi and Iwano 2012; Hobley *et al.* 2013). BslA is synthesized with a 28-amino-acid Sec-dependent signal peptide and, after signal peptide cleavage the 16.4 kDa mature protein is released into the extracellular environment (Ostrowski *et al.* 2011). In the wild-type biofilm, the mature processed BslA can function as a ‘communal good’ benefiting non-producing cells (Ostrowski *et al.* 2011). Experimental evidence is suggestive of BslA stability and localization being dependent on the exopolysaccharide component of the biofilm matrix (Kobayashi and Iwano 2012). Consistent with this, the extreme non-wetting phenotype of the mature biofilm was first correlated with successful production of the large secreted EPS synthesized by the products of the 15-gene *epsA-O* operon. Indeed, the non-wetting nature of the wild-type biofilms is entirely abrogated upon deletion of the *epsA-O* gene cluster (Epstein *et al.* 2011). However, as the *B. subtilis* biofilm EPS is water soluble it seems unlikely that it is directly responsible for the non-wetting phenotype of the mature biofilm. It is possible that the EPS mediates hydrophobicity indirectly as a consequence of currently undefined interactions with BslA. We have previously hypothesized a further possible role of BslA: since the channels through the centre of the biofilm wrinkles allow the passage of liquid and it is possible that BslA may coat these channels allowing for the rapid movement of liquid into the inner regions of the biofilm (Cairns, Hobley and Stanley-Wall 2014). The function of BslA, and the knowledge that the mature BslA protein can be shared with non-producing cells in a mixed co-culture biofilm, supports the definition of *B. subtilis* biofilms as social, cooperative, bacterial communities.

## THE MECHANISM OF BslA ACTIVITY

A partial explanation of how BslA functions was derived from *in vitro* experiments using purified recombinant BslA. Tensiometry demonstrated that the recombinant BslA, while soluble in solution, was surface active meaning that the protein migrates to an air/water or oil/water interface where it forms an elastic film (Hobley *et al.* 2013). The crystal structure of BslA_48–171_ provided atomic level insight to the surface activity and revealed the presence of a highly hydrophobic ‘cap’ domain with nine exposed leucine and isoleucine residues displayed on an immunoglobulin IgG-like scaffold (Hobley *et al.* 2013). Based on these findings, BslA was named a ‘bacterial hydrophobin’ in line with the terminology used to classify the proteins that coat fungal fruiting bodies and aerial hyphae (Elliot and Talbot 2004). It should be noted, however, that BslA does not exhibit sequence or structural similarity to the fungal hydrophobins. It is clear that the analysis of the biophysical and molecular mechanisms underpinning the assembly of matrix components such as BslA will have implications for understanding how to disrupt bacterial biofilms.

## TasA FIBRE FORM AND FORMATION

The production and assembly of amyloid-like fibres in the *B. subtilis* biofilm matrix is accomplished by the proteins encoded within the *tapA-sipW-tasA* operon (Branda *et al.* 2006; Romero *et al.* 2010) (Fig. 1B) (Table 1). TasA is the major protein component of the extracellular fibres and, consistent with this, deletion of *tasA* blocks the formation of a robust, rugose biofilm both *in vitro* and *in planta* (Branda *et al.* 2006; Beauregard *et al.* 2013). Biofilm formation on plant root surfaces has been linked with biocontrol properties exhibited by *B. subtilis* (Bais, Fall and Vivanco 2004) and, reflecting the biofilm defect exhibited by the *tasA* mutant, it confers reduced protection against pathogen attack compared with the wild-type strain (Chen *et al.* 2013). Intriguingly, in laboratory isolates of *B. subtilis*, deletion of *tasA* is not associated with a decrease in surface-adhered biofilm formation suggesting that TasA is not needed for submerged biofilm formation (Branda *et al.* 2004; Hamon *et al.* 2004). The experimental basis for this behaviour remains to be elucidated.

TasA is synthesized with a 27-amino-acid Sec-dependent signal peptide. The 25.7 kD mature protein is released into the extracellular environment after signal peptide cleavage by SipW (Stover and Driks 1999b; Terra *et al.* 2012) (Fig. 1B). Here it is assembled into fibre form in a TapA-dependent manner (Romero *et al.* 2010). The first biological function assigned to TasA (originally called CotN) was as a spore-associated protein that exhibited broad spectrum antimicrobial activity (Stover and Driks 1999b). It has been speculated that the antimicrobial activity associated with TasA may help provide protection to the cells within the biofilm (Romero *et al.* 2010). The propensity of TasA to form fibres was initially discovered using electron microscopy analysis coupled with immunogold-labelling detection techniques (Romero *et al.* 2010). It was also noted that TasA fibres could be purified from the extracellular milieu of *B. subtilis* cultures and that they were able to bind Thioflavin T and Congo red (Romero *et al.* 2010); as discussed above, these characteristics are consistent with (but not evidential of) amyloid fibre formation (Khurana *et al.* 2001; Eisert, Felau and Brown 2006). Secondary structure analysis highlighted a high proportion of β-strand content in the purified protein fibres (Romero *et al.* 2010) (Table 1). After this initial analysis, further biochemical and biophysical experiments indicated that TasA was purified in an oligomeric state in solution (Chai *et al.* 2013) and that fibre formation was stimulated over time *in vitro* by environmental conditions that included hydrophobic surfaces, such as electron microscopy grids (Romero *et al.* 2010) and acidic solutions (Chai *et al.* 2013). As the authors note, these findings raise the fascinating question of what triggers TasA fibre formation in the natural environment of the biofilm. It is interesting to speculate that the hydrophobic properties of the biofilm raincoat protein BslA might be involved.

## FACILITATING FIBRE ASSEMBLY AND ATTACHMENT BY TapA

Assembly of TasA fibres in the biofilm is dependent on TapA (renamed from YqxM for *T*asA *a*nchoring/assembly *p*rotein) (Romero *et al.* 2011). The mature assembled TasA fibres serve as a ‘common good’ in the biofilm matrix that are capable of being shared with non-producing cells (Branda *et al.* 2006). This has been determined using both biochemical and genetic approaches that supply TasA oligomers from the extracellular environment. However, consistent with an intimate working relationship between TasA and TapA during the elaboration of functional TasA fibres from the cell body, synthesis of these two proteins needs to occur in the same cell (Romero *et al.* 2011). For example, co-culture of the *tasA* and *tapA* mutant cell lines does not allow formation of a wild-type biofilm (Romero *et al.* 2011). This is in contrast to the curli subunits CsgA and CsgB produced by *E. coli*, where CsgB acts as a nucleator (like TapA) and CsgA is the major fibre component (like TasA). In this system, CsgA and CsgB can be produced by separate cells and CsgB will still act as a nucleator and facilitate polymerization of CsgA into fibres in the exogenous environment (Chapman *et al.* 2002). It will be of interest to understand the molecular reasons for this difference.

TapA is a 253-amino-acid protein that is synthesized with a 33-amino-acid Sec-dependent signal sequence cleaved upon secretion by SipW, a dedicated signal peptidase (Stover and Driks 1999a). TapA is predominantly found anchored to the cell wall but also forms a minor component (1:100) of the TasA fibres (Romero *et al.* 2011, 2014) (Fig. 1B). There is very little molecular data regarding TapA function, and primary sequence bioinformatics analysis yields limited information. However, the presence of five conserved cysteine residues and of two regions each containing two imperfect repeat sequences was recently noted (Romero *et al.* 2014). The cysteine residues were shown only to have a limited impact on TapA function; in contrast, disruption of the N-terminal imperfect repeat by either deletion or replacement of amino acids 50–57 (TFDVSLQT) impaired biofilm formation, although the protein was still seen to localize on the cell surface. Furthermore, the variant TapA protein displayed a dominant negative effect on wild-type TapA function, and inhibited TasA polymerization *in vitro* (Romero *et al.* 2014). It is possible that the deletion of the first N-terminal repeat inhibits TasA polymerization, but does not inhibit either TapA cell-surface localization or incorporation into the fibres, thereby inhibiting the onset of fibre polymerization *in vivo* and also halting *in vitro* fibre formation at high ratios of TapA to TasA. It is clear that while our understanding of TasA/TapA function is growing, our knowledge base would strongly benefit from atomic-level structural data that may help to inform the existence of any interactions that exist between the two proteins and indeed the other matrix molecules. Furthermore, the ability to form biofilms in the laboratory has been correlated with the ability of some *B. subtilis* isolates to persist within the gastrointestinal tract of mice, and whilst a definitive cause and effect has not yet been shown it has been hypothesized that biofilm formation may be responsible for the persistence of germinated spores after ingestion (Tam *et al.* 2006). It will be of interest to understand how (and if) the extracellular macromolecules interact with the host gut environment and commensal intestinal flora.

## STAPHYLOCOCCUS AUREUS

*Staphylococcus aureus* is a major human and live-stock-associated pathogen that is capable of causing diseases ranging from superficial skin infections to life-threatening sepsis. To achieve this, the organism has acquired diverse mechanisms to colonize and evade the host immune response. Of these mechanisms, the formation of biofilms is particularly problematic given the impermeability of the bacterial communities to the host immune cells and antibiotic therapy (Costerton, Stewart and Greenberg 1999). *Staphylococcus aureus* biofilm infections have been directly linked with infective endocarditis or implant-associated infection, causing persistent and destructive diseases which are a massive burden in respects to both morbidity and mortality (Costerton, Stewart and Greenberg 1999; Jones *et al.* 2001). Both clinically and in the laboratory, the molecules that aid biofilm formation by *S. aureus* are strain-specific and, although the mechanism remains unclear, are noted to vary between methicillin-sensitive and methicillin-resistant (MSSA/MRSA) lineages (O'Neill *et al.* 2007; Geoghegan *et al.* 2013).

Broadly speaking, Staphylococcal biofilm accumulation can be viewed as: *ica*-dependent, relying upon polysaccharide intercellular adhesion production; dependent on the high molecular weight poly-N-acetyl-β-(1–6)-glucosamine (PNAG) which is synthesized by the products of the *icaADBC* operon (Cramton *et al.* 1999; Maira-Litran *et al.* 2002); or *ica*-independent. Indeed, while the *ica* operon is present in most *S. aureus* isolates its expression varies in a strain and growth condition-dependent manner (Cramton *et al.* 2001). Transcription of the *icaADBC* operon has been shown to be controlled by phase variation, with PNAG-negative strains identified as possessing a growth advantage over the PNAG-producing siblings (Ziebuhr *et al.* 1997, 1999; Brooks and Jefferson 2014). Upon production of the PNAG polymer, in addition to aiding biofilm formation, the bacteria gain protection from the host immune system, thereby attaining an additional growth advantage (Cerca *et al.* 2007).

There are currently two models for PNAG biosynthesis and secretion by *S. aureus* (Atkin *et al.* 2014); the first is as follows: IcaA as an GlcNAc transferase needed for the synthesis of the PNAG polymer where the function of IcaA is dependent on the integral membrane protein IcaD (Gerke *et al.* 1998). IcaB is an N-deacetylase that is specific to PNAG (Pokrovskaya *et al.* 2013). This leaves IcaC, which is an integral membrane protein with multiple transmembrane domains that is thought to play a direct role in PNAG export. The second, more recently, postulated mechanism of PNAG biosynthesis and export places an IcaAD membrane complex responsible for the export of PNAG with IcaC functioning to add succinyl groups to the growing polymer using its *O*-succinyltransferase activity (Atkin *et al.* 2014). Either way, a greater understanding of the biosynthesis and structure of PNAG is likely to be of therapeutic benefit during the treatment of ICA-dependent biofilm infections. In *ica*-independent biofilms, proteinaceous factors are utilized to allow biofilm development, a phenotype which appears to be more prevalent in MRSA isolates (O'Neill *et al.* 2007). Here, we focus on the cell-wall-anchored (CWA) and secreted proteins that promote biofilm formation by this important pathogenic species of bacteria.

## CELL WALL ANCHORED PROTEINS

**Figure 5.**
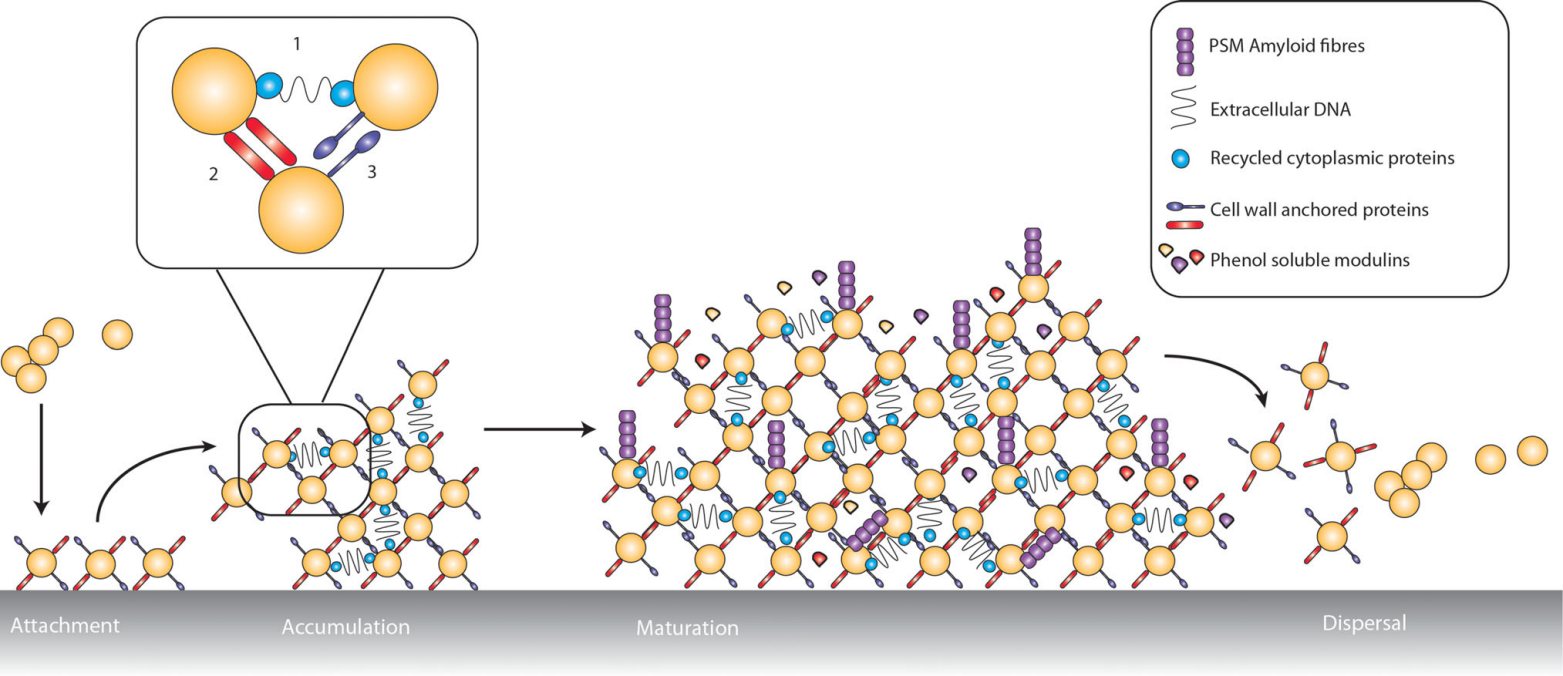
*Staphylococcus aureus* biofilm formation. Attachment of *S. aureus* to a surface is mediated by CWA proteins. Cell-to-cell interactions occur during accumulation phase and can be mediated by several factors. The magnified region shows this in more detail: (**1)** extracellular DNA linking recycled cytoplasmic proteins; (**2)** CWA proteins binding adjacent cell surfaces; (**3)** Homophilic interactions between CWA proteins. PSMs form amyloid-like fibres visible at the surface of the biofilm. They also act in the formation of channels within the biofilm to allow nutrient access, while their surfactant properties aid the dispersal phase. The different stages of biofilm formation are detailed from left to right across the diagram.

**Figure 6.**
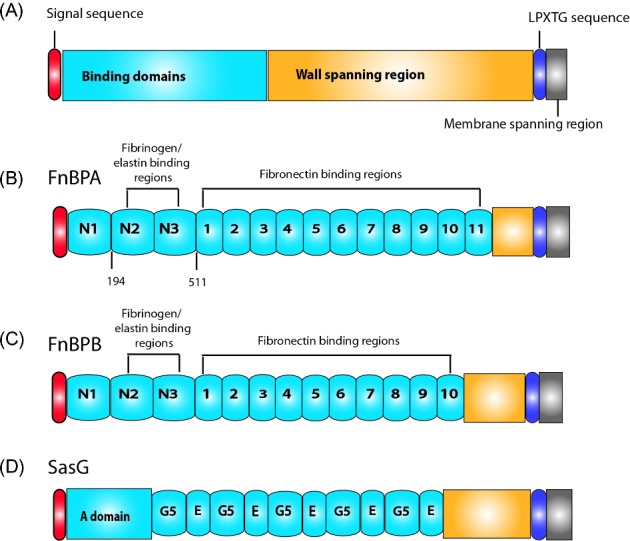
Structure of the CWA proteins. All CWA proteins contain a Sec-dependent secretory signal sequence, a C-terminal LPXTG sortase motif, a hydrophobic domain and finally a chain of positively charged residues at the end of the C-terminus. **(A)** The schematic demonstrates the typical domain structure within the MSCRAMM family of CWAs. At the N-terminus, a Sec-dependent signal sequence followed by a variable number of binding domains that begin with an N-terminal A domain (inclusive of N subdomains in the case of FnBPs (**B and C**) and ClfB). These are followed by a wall-spanning region, the LPXTG motif and finally a membrane-spanning region. **(D)** In SasG, the binding domain is subdivided into the N-terminal A domain and a varying number of G5/E repeats.

In *S. aureus*, the expression of CWA proteins is integral to the ability of the organism to attach to a surface and thereby initiate biofilm formation (Fig. 5). At the molecular level, CWA proteins are characterized by the presence of a Sec-dependent secretory signal sequence at the N-terminus and contain an ‘LPXTG’ motif at the C-terminus (Fig. 6A). Upon secretion, the proteins are cleaved by Sortase A (SrtA), a membrane-bound transpeptidase, which catalyses the attachment of the processed protein to the cell wall peptidoglycan (Ton-That *et al.* 1999). *S. aureus* encodes up to 24 different CWAs and there can be significant variation in expression between strains and in a growth condition-specific manner (Foster *et al.* 2014). For example, strain ‘Newman’ is a clinical isolate that forms weak biofilms, most likely as it carries mutations in the coding regions for the Fnbp proteins (*vide infra*) (Grundmeier *et al.* 2004). To date, the CWA family of proteins has been the most extensively studied with respect to their function as biofilm-associated factors that bind ligands on cell surfaces to allow adhesion.

The CWA proteins that have been demonstrated to be of importance during *in vitro* and *in vivo* biofilm development include the biofilm-associated protein (Bap) (Cucarella *et al.* 2001), the fibronectin-binding proteins A/B (FnbpA/B) (O'Neill *et al.* 2008; Geoghegan *et al.* 2013), clumping factor B (ClfB) (Abraham and Jefferson 2012), serine-aspartate repeat protein C (SdrC) (Barbu *et al.* 2014), *S. aureus* surface protein C (SasC) (Schroeder *et al.* 2009), *S. aureus* surface protein G (SasG) (Geoghegan *et al.* 2010) and protein A (Merino *et al.* 2009). It should be noted that each of these proteins, with the exception of Bap, has been detected in human clinical isolates, while Bap has been found in bovine isolates only (Cucarella *et al.* 2001). The proteins bind to eukaryotic extracellular matrix proteins, or to surfaces which have been primed by host plasma such as medical implants (Lower *et al.* 2011).

## LIGANDS OF THE CWA PROTEINS

Several of the CWA proteins have known ligands, and it has been shown that they can have overlapping targets or in fact more than one target for binding. For instance, the clumping factors A and B (ClfA/B) (Deivanayagam *et al.* 2002; Ganesh *et al.* 2011) and fibronectin-binding proteins A/B (FnbpA/B) each bind the plasma protein fibrinogen in a specific manner (Wann, Gurusiddappa and Hook 2000; Burke *et al.* 2011). Additionally, a number of the CWAs have ligands which are epidermal proteins. For example, ClfB is known to bind the epithelial cell envelope protein Loricrin aiding nasal colonization (Mulcahy *et al.* 2012), while iron-regulated surface protein A (IsdA) binds to cytokeratin 10 in desquamated human epithelial cells (Clarke *et al.* 2009). It is conceivable that the functional overlap with respect to ligand binding has developed as a means to maximize the chances of *S. aureus* adherence. Selective pressures within the host have also presumably driven the development of body site-specific ligands, e.g. Loricrin or Cytokeratin, as a way of enabling attachment. These processes of adaptation within the CWA proteins appear to be continual, and may play a role in the outcome of infection. For instance, it has been shown that bacterial isolates extracted from cardiac device implants have single amino acid polymorphisms within the fibronectin-binding domains. These amino acid level changes result in higher ligand-binding affinity to fibronectin than found in isolates from bacteraemia or nasal isolates (Lower *et al.* 2011).

While CWAs can be classified on the basis of structure, detail regarding the molecular mechanism by which *S. aureus* uses CWA proteins during biofilm formation remains limited in many cases. For instance, Protein A has been classed structurally as a three helical bundle protein, containing five homologous IgG-binding domains (Graille *et al.* 2000). When it is produced at high levels on the cell surface, Protein A is able to promote biofilm formation, but *how* aggregation of the cells is triggered is unknown (Merino *et al.* 2009). Other CWAs remain structurally uncharacterized, for example SasC which has been shown to contribute to intercellular adhesion and biofilm formation (Schroeder *et al.* 2009), although its role at the molecular level remains to be defined. A common theme is that lack of insight of the CWA protein ligands is limiting our understanding of their function. Use of techniques such as phage display has elucidated that SdrC (classified as a MSCRAMM, see below) homophilic interactions in *S. aureus* are a mode of cell aggregation in biofilm formation (Barbu *et al.* 2014). This technique could be a prospective means for identifying new ligands for other CWA proteins, to allow inroads to potential routes of blocking/disrupting intercellular interaction and biofilm formation.

## RELATING STRUCTURE TO FUNCTION IN THE CWA PROTEINS

CWA proteins can be subdivided into categories according to their structural and ligand-binding features. Many CWA proteins bind to the extracellular matrix and these proteins have generally been termed *M*icrobial *S*urface *C*omponents *R*ecognizing Surface *A*dhesive *M*atrix *M*olecules (MSCRAMMs). It has recently been proposed that this acronym be used to specifically describe a family of surface proteins which share structural and ligand-binding features (Foster *et al.* 2014) (Fig. 5). First, we will consider the MSCRAMMs that include the proteins ClfA/B, FnbpA/B and SdrC among others (Foster *et al.* 2014). They are characterized by an N-terminal signal sequence (Sec), N-terminal A domain with 2 or more IgG-like domains (Deivanayagam *et al.* 2002) and a C-terminal LPXTG sortase recognition sequence (Fig. 6B and C). ClfA is the classically used example of an MSCRAMM protein. The fibrinogen-binding domain A of ClfA has been characterized in crystal form, and based upon this, structural predictions suggest that the other MSCRAMMs have highly similar A domain structures (Deivanayagam *et al.* 2002). The fibrinogen and fibronectin-binding capability of ClfA has been localized to the N-terminal A domains (Hartford *et al.* 2001). This binding capability allows *S. aureus* to adhere to the extracellular matrix to initiate biofilm formation. For the Fnbp's, functional assessment of the A domains has demonstrated that the fibrinogen-binding A subdomain is sufficient to promote biofilm formation, with the fibronectin-binding domains being dispensable (O'Neill *et al.* 2008). Further analysis of mutations within the N1, N2 and N3 subdomains of the A domain have demonstrated that N3 is required for FnbpA to bind to fibrinogen, whilst N2 and N3 mediate accumulation of the biofilm (Fig. 6B and C). It remains mechanistically unclear how Fnbp proteins mediate accumulation, but is hypothesized to involve homophilic interaction of N2–N3 domains on sibling bacteria (Geoghegan *et al.* 2013). The MSCRAMM ClfB is known to bind fibrinogen, but this function is distinct from its ability to mediate cell aggregation which appears to be calcium dependent (Abraham and Jefferson 2012). It will be necessary to identify the binding sites responsible for these proposed cell-to-cell interactions, not least as they could provide potential means of disrupting and clearing biofilms mediated by MSCRAMMs.

## SasG, A FIBRE FORMING CWA PROTEIN

As detailed above for *E. coli* and *B. subtilis*, biofilm formation by *S. aureus* requires the production of fibre-forming proteins. SasG is a CWA protein that aids binding of bacterial cells to desquamated nasal epithelial cells (Roche, Meehan and Foster 2003). Structurally SasG is classified as a member of the GE repeat family (Foster *et al.* 2014). It forms (non-amyloid) β-sheet-rich protein fibrils that protrude from the cell surface, which can be visualized by electron microscopy (Corrigan *et al.* 2007). The SasG fibrils have been proposed to represent a mechanism by which individual bacteria can become linked together during the attachment phase of biofilm formation (Geoghegan *et al.* 2010; Foster *et al.* 2014). With respect to protein domain organization, SasG follows the typical pattern of the LPXTG proteins where, following the N-terminal A domain in the mature protein, there are multiple tandem GE repeats (Fig. 6D). Within these repeats, sequences contain G5 domains of approximately 80 residues, which are followed by 50 residue sequences known as E regions (Gruszka *et al.* 2012) (Fig. 6D). It is specifically the GE repeat regions of the SasG protein that have been identified as being required for the accumulation of biofilms (Geoghegan *et al.* 2010). Mechanistic insight to SasG function has been derived from analysis of the crystal structure of the EG5 domains which was found to be composed of a single layer of β-sheets. Interlocking connections between the E and G5 domains of SasG leads to the formation of rod-like protein structures which are responsible for protein fibrils on the cell surface (Gruszka *et al.* 2012).

## PHENOL SOLUBLE MODULINS

A group of peptides that have been recently discovered to be of importance in both the development and stabilization of biofilms formed by *S. aureus* are phenol soluble modulins (PSMs) (Table 1) (Figs 1C and 5). Discovered originally in *S. epidermidis* as immunomodulatory peptides, it is now well accepted that the proteins confer virulence to *S. aureus* (Cheung *et al.* 2014). These small peptides have an α-helical amphipathic structure which is responsible for the surfactant-type properties they display in their monomeric state (Wang *et al.* 2011; Periasamy *et al.* 2012). The proteins are highly conserved across *S. aureus* strains, and are encoded for at three distinct regions within the genome. The regions include the alpha operon which expresses *αpsm*1–4, the beta operon expressing β1 and 2, and *RNAIII* which is a regulatory RNA responsible for the expression of delta hemolysin (Mehlin, Headley and Klebanoff 1999; Wang *et al.* 2007). Transcription of all three operons is under the control of the accessory gene regulator (Agr) system (Queck *et al.* 2008).

The role of PSMs during biofilm formation was assessed by direct comparisons of wild-type biofilm formation with that of the *psm* and *agr* mutants. Analysis demonstrated that PSMs are essential at multiple levels and that their absence prevented the normal maturation of the biofilm. More specifically, PSMs are required for structuring of the biofilm where they have direct involvement in the formation of channels through which nutrients can be obtained, dissemination of cells from the biofilm, and expansion of the biofilm (Periasamy *et al.* 2012). PSMβ peptides appear to have the most pronounced impact on biofilm structuring (Periasamy *et al.* 2012). These findings are particularly interesting if we consider the prolific biofilm-forming *S. epidermidis.* Although capable of producing all of the PSMs, *S. epidermidis* predominantly produces the poorly cytolytic PSMβ in culture (Wang *et al.* 2011). It may therefore be the case that PSMβ has evolved specifically to function in structuring of the biofilm.

As with many virulence factors in Staphylococci, PSM production is dependent on both nutrient availability and growth state (Schwartz *et al.* 2012). It was through investigation of the effect of growth media on biofilm matrix composition that led to the discovery that PSMs form fibrillar structures in *S. aureus* biofilms (Schwartz *et al.* 2012). Growth of *S. aureus* in peptone–NaCl–glucose media produced biofilms resistant to dispersal. In these biofilms, hair-like fibres were visible using transmission electron microscopy, but notably absent in *agr/*Δαβpsm mutants that were prone to dispersal. Intact fibres conformed to traits attributed to amyloid proteins including SDS insolubility, staining with amyloid-specific markers and β-sheet structure (Gebbink *et al.* 2005). Subsequent purification of the fibres revealed several peptides covering the PSM family. Current thinking is that the formation of these fibrils is a means of storing toxic PSMs until the additional functions as antimicrobials and virulence factors are required (Schwartz *et al.* 2012). As the most recently described group of biofilm-associated peptides/fibres, many aspects of their biology remain to be uncovered. Further elucidation of the process by which PSMs form amyloid-like fibres and if they exist *in vivo* will be important to establish. Additionally, given the cytolytic properties of the PSMs, which lead to bacterial cell death, and their specific export system (Chatterjee *et al.* 2013) (Fig. 1C), inhibition of export is an attractive prospective area of study in the search for anti-infective agents. Whether small molecules targeted towards this system could be harnessed as a possible means to destroy Staphylococci during biofilm formation/infection remains to be established.

## THE MATRIX OF *S. AUREUS* BIOFILMS

Emerging as additional possible components of the extracellular matrix of *S. aureus* biofilms are recycled cytoplasmic proteins (Foulston *et al.* 2014). Using a proteomics approach, cell surface proteins derived from biofilm (^14^N) or non-biofilm (^15^N) growth conditions were biotinylated and analysed comparatively. A total of 11 proteins were found to be significantly enriched in biofilm growth conditions, all of which were predicted cytoplasmic proteins with functions in metabolism. Through multiple approaches, including live/dead cell staining, it was demonstrated that these cytoplasmic proteins were released from live cells during the development of the biofilm and remained cell associated. With SDS-PAGE analysis, it was shown that the proteins are retained at the cell surface of biofilm cells at a pH of 5, and reversibly released when the pH increased again. The pH-dependent mechanism of secretion of these cytoplasmic proteins has not been elucidated; however, the authors hypothesized that the cytoplasmic proteins coating the cell surface could carry a net positive charge which would act to link to eDNA, and contribute to the biofilm matrix (Foulston *et al.* 2014). These findings provide a new and interesting mechanism by which an organism can adapt to form a multicellular community depending on environmental conditions. Further interrogation of these mechanisms is certainly justified given that hyperglycaemic states (and thus low pH) are both a risk and a feature of chronic biofilm infections such as diabetic ulcers (James *et al.* 2008).

## EMERGING TRENDS AND FUTURE DEVELOPMENT AREAS

The recognition of biofilms as the major reservoir of bacteria within the natural environment, and the subsequent realization of the importance of biofilm formation to the survival and pathogenicity of a vast number of bacterial species has spurred the growth in interest and research into the molecular mechanisms underlying biofilm formation. The focus on a select group of model organisms has revealed several conserved themes that appear to underlie biofilm formation in all species. The first and foremost is that the biofilm matrix is not simply a ‘slime’ that surrounds the cells. It is in fact a highly ordered structure, with extensive amounts of protein localization, and interactions between components, that result in a robust and protective biofilm ‘coat’. The structural components of the matrix are predominantly a mixture of polysaccharides and proteins, and many of these proteins have been shown to polymerize into higher-order structures. For example, the curli fibres made by *E. coli* and other Enterobacteria, the TasA fibres and bacterial hydrophobin BslA, both produced by *B. subtilis*, and the PSM and the SasG β-sheet-rich fibres of *S. aureus* are all ordered protein aggregates. It is interesting to speculate that one of the proteins required for biofilm formation by *V. cholerae* may be found to form higher-order structures in the future.

The adaptation of various immunofluorescence microscopy techniques has been used to study many of the biofilms discussed in this review. For instance, curli and cellulose localization in *E. coli* biofilms was analysed by staining the curli and cellulose with Thioflavin S (Serra, Richter and Hengge 2013a), the localization of the hydrophobin ‘raincoat’ protein BslA in *Bacillus* biofilms was studied using antibodies specific to BslA combined with fluorescent secondary antibodies prior to confocal microscopy (Hobley *et al.* 2013) and the localization of the protein components of the *V. cholerae* biofilm was studied using high-resolution four-colour immunofluorescence confocal microscopy (Berk *et al.* 2012). For the first two techniques, cryosectioning of the biofilms was used prior to imaging, allowing for analysis of only a single point in the course of biofilm formation. In contrast, the final technique was adapted for use in real time, such that the founding bacterial cells could be tracked during the course of formation. With advances in microscopy, it is probable that imaging within a growing colony biofilm may soon be possible, in a manner similar to that already employed for the submerged surface-attached *Vibrio* biofilms. Super-resolution microscopes also exist such that single molecules within a sample can be imaged. Applying this form of microscopy to the analysis of both the protein and polysaccharide components of the matrix (for example using fluorescently labelled amino acids or sugars) may reveal further detail as to how these two major matrix components interact and provide the incredible structural integrity to the biofilm. Similarly, the advances in *in situ* NMR-based approaches may also reveal further information about the structure of the polysaccharide components as well as revealing the quantitative ratios of each component.

By ultimately understanding the mechanisms by which the biofilm builds and maintains its structural integrity, the biofilm matrix may become the target of future antimicrobial drug design. Biofilms are known to be a harbour for many antibiotic-resistant pathogens, and it is only by targeting the biofilm that the most effective drugs will be produced. For instance, significant effort is directed at the development of novel vaccines for *S. aureus* through utilising the fundamental knowledge of the polysaccharide and adhesins needed for biofilm establishment (Jansen *et al.* 2013). It is however important to keep in mind that not all biofilm-forming bacteria are pathogenic species. Biofilm formation is known to be essential for some beneficial functions, for example *B. subtilis* can act as a plant protectant agent when it grows on plant roots (Chen *et al.* 2013; Gao *et al.* 2013). When colonizing plant roots, biofilm formation is essential for efficient surface coverage, and the protein matrix component TasA and the exopolysaccharide have both been shown to be essential for effective plant root colonisation in both Arabidopsis and Tomato plants (Rudrappa *et al.* 2007; Beauregard *et al.* 2013; Chen *et al.* 2013). It is possible that by further understanding biofilm formation by such species, we can design treatments that actually promote or enhance biofilm formation on the plant root, leading to a more effective plant protectant agent. Given that biofilm research is still in its (comparative) infancy, when compared with the study of planktonically grown cells, our advances in understanding the complex mechanisms that result in the formation of these multicellular communities have been immense. The advances in microscopy and spectroscopy techniques and the merging of expertise between disciplines that is currently underway make the future of biofilm research very exciting!

## References

[bib1] Abraham NM, Jefferson KK (2012). *Staphylococcus aureus* clumping factor B mediates biofilm formation in the absence of calcium. Microbiology.

[bib2] Absalon C, Van Dellen K, Watnick PI (2011). A communal bacterial adhesin anchors biofilm and bystander cells to surfaces. PLoS Pathog.

[bib3] Altindis E, Fu Y, Mekalanos JJ (2014). Proteomic analysis of *Vibrio cholerae* outer membrane vesicles. P Natl Acad Sci USA.

[bib4] Andersson EK, Bengtsson C, Evans ML (2013). Modulation of curli assembly and pellicle biofilm formation by chemical and protein chaperones. Chem Biol.

[bib5] Asally M, Kittisopikul M, Rue P (2012). Localized cell death focuses mechanical forces during 3D patterning in a biofilm. P Natl Acad Sci USA.

[bib6] Atkin KE, MacDonald SJ, Brentnall AS (2014). A different path: revealing the function of staphylococcal proteins in biofilm formation. FEBS Lett.

[bib7] Bais HP, Fall R, Vivanco JM (2004). Biocontrol of *Bacillus subtilis* against infection of Arabidopsis roots by *Pseudomonas syringae* is facilitated by biofilm formation and surfactin production. Plant Physiol.

[bib8] Barbu EM, Mackenzie C, Foster TJ (2014). SdrC induces staphylococcal biofilm formation through a homophilic interaction. Mol Microbiol.

[bib9] Barnhart MM, Chapman MR (2006). Curli biogenesis and function. Ann Rev Microbiol.

[bib10] Beauregard PB, Chai Y, Vlamakis H (2013). *Bacillus subtilis* biofilm induction by plant polysaccharides. P Natl Acad Sci USA.

[bib11] Ben Nasr A, Olsen A, Sjobring U (1996). Assembly of human contact phase proteins and release of bradykinin at the surface of curli-expressing *Escherichia coli*. Mol Microbiol.

[bib12] Benz I, Schmidt MA (2001). Glycosylation with heptose residues mediated by the *aah* gene product is essential for adherence of the AIDA-I adhesin. Mol Microbiol.

[bib13] Berk V, Fong JC, Dempsey GT (2012). Molecular architecture and assembly principles of *Vibrio cholerae* biofilms. Science.

[bib14] Bian Z, Normark S (1997). Nucleator function of CsgB for the assembly of adhesive surface organelles in *Escherichia coli*. EMBO J.

[bib15] Borlee BR, Goldman AD, Murakami K (2010). *Pseudomonas aeruginosa* uses a cyclic-di-GMP-regulated adhesin to reinforce the biofilm extracellular matrix. Mol Microbiol.

[bib16] Branda SS, Chu F, Kearns DB (2006). A major protein component of the *Bacillus subtilis* biofilm matrix. Mol Microbiol.

[bib17] Branda SS, Gonzalez-Pastor JE, Ben-Yehuda S (2001). Fruiting body formation by *Bacillus subtilis*. P Natl Acad Sci USA.

[bib18] Branda SS, Gonzalez-Pastor JE, Dervyn E (2004). Genes involved in formation of structured multicellular communities by *Bacillus subtilis*. J Bacteriol.

[bib19] Branda SS, Vik S, Friedman L (2005). Biofilms: the matrix revisited. Trends Microbiol.

[bib20] Brooks JL, Jefferson KK (2014). Phase variation of poly-N-acetylglucosamine expression in *Staphylococcus aureus*. PLoS Pathog.

[bib21] Burke FM, DiPoto A, Speziale P (2011). The A domain of fibronectin-binding protein B of *Staphylococcus aureus* contains a novel fibronectin binding site. FEBS J.

[bib22] Cairns LS, Hobley L, Stanley-Wall NR (2014). Biofilm formation by *Bacillus subtilis*: new insights into regulatory strategies and assembly mechanisms. Mol Microbiol.

[bib23] Cao B, Zhao Y, Kou Y (2014). Structure of the nonameric bacterial amyloid secretion channel. P Natl Acad Sci USA.

[bib24] Cegelski L, Pinkner JS, Hammer ND (2009). Small-molecule inhibitors target *Escherichia coli* amyloid biogenesis and biofilm formation. Nat Chem Biol.

[bib25] Cerca N, Jefferson KK, Maira-Litran T (2007). Molecular basis for preferential protective efficacy of antibodies directed to the poorly acetylated form of staphylococcal poly-N-acetyl-beta-(1–6)-glucosamine. Infect Immun.

[bib26] Chai L, Romero D, Kayatekin C (2013). Isolation, characterization, and aggregation of a structured bacterial matrix precursor. J Biol Chem.

[bib27] Chai Y, Beauregard PB, Vlamakis H (2012). Galactose metabolism plays a crucial role in biofilm formation by *Bacillus subtilis*. MBio.

[bib28] Chapman MR, Robinson LS, Pinkner JS (2002). Role of *Escherichia coli* curli operons in directing amyloid fiber formation. Science.

[bib29] Chatterjee SS, Joo HS, Duong AC (2013). Essential *Staphylococcus aureus* toxin export system. Nat Med.

[bib30] Chauhan A, Sakamoto C, Ghigo JM (2013). Did I pick the right colony? Pitfalls in the study of regulation of the phase variable antigen 43 adhesin. PloS One.

[bib31] Chen Y, Yan F, Chai Y (2013). Biocontrol of tomato wilt disease by *Bacillus subtilis* isolates from natural environments depends on conserved genes mediating biofilm formation. Environ Microbiol.

[bib32] Cheung GY, Joo HS, Chatterjee SS (2014). Phenol-soluble modulins–critical determinants of staphylococcal virulence. FEMS Microbiol Rev.

[bib33] Clarke SR, Andre G, Walsh EJ (2009). Iron-regulated surface determinant protein A mediates adhesion of *Staphylococcus aureus* to human corneocyte envelope proteins. Infect Immun.

[bib34] Clements A, Young JC, Constantinou N (2012). Infection strategies of enteric pathogenic *Escherichia coli*. Gut Microbes.

[bib35] Cohn F (1877). Untersuchungen uber Bacterien. IV. Beitrage zur Biologie der Bacillen. Beitr Biol Pflanz.

[bib36] Collinson SK, Clouthier SC, Doran JL (1996). *Salmonella enteritidis agfBAC* operon encoding thin, aggregative fimbriae. J Bacteriol.

[bib37] Collinson SK, Emody L, Muller KH (1991). Purification and characterization of thin, aggregative fimbriae from *Salmonella enteritidis*. J Bacteriol.

[bib38] Collinson SK, Parker JM, Hodges RS (1999). Structural predictions of AgfA, the insoluble fimbrial subunit of Salmonella thin aggregative fimbriae. J Mol Biol.

[bib39] Colwell RR, Huq A, Islam MS (2003). Reduction of cholera in Bangladeshi villages by simple filtration. P Natl Acad Sci USA.

[bib40] Corrigan RM, Rigby D, Handley P (2007). The role of *Staphylococcus aureus* surface protein SasG in adherence and biofilm formation. Microbiology.

[bib41] Costerton JW, Cheng KJ, Geesey GG (1987). Bacterial biofilms in nature and disease. Annu Rev Microbiol.

[bib42] Costerton JW, Stewart PS, Greenberg EP (1999). Bacterial biofilms: a common cause of persistent infections. Science.

[bib43] Cramton SE, Gerke C, Schnell NF (1999). The intercellular adhesion (*ica*) locus is present in *Staphylococcus aureus* and is required for biofilm formation. Infect Immun.

[bib44] Cramton SE, Ulrich M, Gotz F (2001). Anaerobic conditions induce expression of polysaccharide intercellular adhesin in *Staphylococcus aureus* and *Staphylococcus epidermidis*. Infect Immun.

[bib45] Cucarella C, Solano C, Valle J (2001). Bap, a *Staphylococcus aureus* surface protein involved in biofilm formation. J Bacteriol.

[bib46] Danese PN, Pratt LA, Dove SL (2000). The outer membrane protein, antigen 43, mediates cell-to-cell interactions within *Escherichia coli* biofilms. Mol Microbiol.

[bib47] Davey ME, O'Toole GA (2000). Microbial biofilms: from ecology to molecular genetics. Microbiol Mol Biol R.

[bib48] Deivanayagam CC, Wann ER, Chen W (2002). A novel variant of the immunoglobulin fold in surface adhesins of *Staphylococcus aureus*: crystal structure of the fibrinogen-binding MSCRAMM, clumping factor A. EMBO J.

[bib49] DePas WH, Syed AK, Sifuentes M (2014). Biofilm formation protects Escherichia coli against killing by *Caenorhabditis elegans* and *Myxococcus xanthus*. Appl Environ Microb.

[bib50] Dueholm MS, Albertsen M, Otzen D (2012). Curli functional amyloid systems are phylogenetically widespread and display large diversity in operon and protein structure. PloS One.

[bib51] Dueholm MS, Petersen SV, Sonderkaer M (2010). Functional amyloid in *Pseudomonas*. Mol Microbiol.

[bib52] Dueholm MS, Sondergaard MT, Nilsson M (2013). Expression of Fap amyloids in *Pseudomonas aeruginosa, P. fluorescens*, and *P. putida* results in aggregation and increased biofilm formation. Microbiologyopen.

[bib53] Duperthuy M, Sjostrom AE, Sabharwal D (2013). Role of *the Vibrio cholerae* matrix protein Bap1 in cross-resistance to antimicrobial peptides. PLoS Pathog.

[bib54] Eisert R, Felau L, Brown LR (2006). Methods for enhancing the accuracy and reproducibility of Congo red and thioflavin T assays. Anal Biochem.

[bib55] Elliot MA, Talbot NJ (2004). Building filaments in the air: aerial morphogenesis in bacteria and fungi. Curr Opin Microbiol.

[bib56] Epstein AK, Pokroy B, Seminara A (2011). Bacterial biofilm shows persistent resistance to liquid wetting and gas penetration. P Natl Acad Sci USA.

[bib57] Epstein EA, Reizian MA, Chapman MR (2009). Spatial clustering of the curlin secretion lipoprotein requires curli fiber assembly. J Bacteriol.

[bib58] Faruque SM, Biswas K, Udden SM (2006). Transmissibility of cholera: in vivo-formed biofilms and their relationship to infectivity and persistence in the environment. P Natl Acad Sci USA.

[bib59] Flemming HC, Wingender J (2010). The biofilm matrix. Nat Rev Microbiol.

[bib60] Fong JC, Karplus K, Schoolnik GK (2006). Identification and characterization of RbmA, a novel protein required for the development of rugose colony morphology and biofilm structure in *Vibrio cholerae*. J Bacteriol.

[bib61] Fong JC, Syed KA, Klose KE (2010). Role of Vibrio polysaccharide (*vps*) genes in VPS production, biofilm formation and *Vibrio cholerae* pathogenesis. Microbiology.

[bib62] Foster TJ, Geoghegan JA, Ganesh VK (2014). Adhesion, invasion and evasion: the many functions of the surface proteins of *Staphylococcus aureus*. Nat Rev Microbiol.

[bib63] Foulston L, Elsholz AK, DeFrancesco AS (2014). The extracellular matrix of *Staphylococcus aureus* biofilms comprises cytoplasmic proteins that associate with the cell surface in response to decreasing pH. mBio.

[bib64] Ganesh VK, Barbu EM, Deivanayagam CC (2011). Structural and biochemical characterization of *Staphylococcus aureus* clumping factor B/ligand interactions. J Biol Chem.

[bib65] Gao S, Wu H, Wang W (2013). Efficient colonization and hairpins mediated enhancement in growth and biocontrol of wilt disease in tomato by *Bacillus subtilis*. Lett Appl Microbiol.

[bib66] Gebbink MF, Claessen D, Bouma B (2005). Amyloids–a functional coat for microorganisms. Nature reviews. Microbiology.

[bib67] Geoghegan JA, Corrigan RM, Gruszka DT (2010). Role of surface protein SasG in biofilm formation by *Staphylococcus aureus*. J Bacteriol.

[bib68] Geoghegan JA, Monk IR, O'Gara JP (2013). Subdomains N2N3 of fibronectin binding protein A mediate *Staphylococcus aureus* biofilm formation and adherence to fibrinogen using distinct mechanisms. J Bacteriol.

[bib69] Gerke C, Kraft A, Sussmuth R (1998). Characterization of the N-acetylglucosaminyltransferase activity involved in the biosynthesis of the *Staphylococcus epidermidis* polysaccharide intercellular adhesin. J Biol Chem.

[bib70] Gibson DL, White AP, Rajotte CM (2007). AgfC and AgfE facilitate extracellular thin aggregative fimbriae synthesis in *Salmonella enteritidis*. Microbiology.

[bib71] Giglio KM, Fong JC, Yildiz FH (2013). Structural basis for biofilm formation via the *Vibrio cholerae* matrix protein RbmA. J Bacteriol.

[bib72] Goyal P, Krasteva PV, Van Gerven N (2014). Structural and mechanistic insights into the bacterial amyloid secretion channel CsgG. Nature.

[bib73] Graille M, Stura EA, Corper AL (2000). Crystal structure of a *Staphylococcus aureus* protein A domain complexed with the Fab fragment of a human IgM antibody: structural basis for recognition of B-cell receptors and superantigen activity. P Natl Acad Sci USA.

[bib74] Grundmeier M, Hussain M, Becker P (2004). Truncation of fibronectin-binding proteins in *Staphylococcus aureus* strain Newman leads to deficient adherence and host cell invasion due to loss of the cell wall anchor function. Infect Immun.

[bib75] Gruszka DT, Wojdyla JA, Bingham RJ (2012). Staphylococcal biofilm-forming protein has a contiguous rod-like structure. P Natl Acad Sci USA.

[bib76] Gualdi L, Tagliabue L, Bertagnoli S (2008). Cellulose modulates biofilm formation by counteracting curli-mediated colonization of solid surfaces in *Escherichia coli*. Microbiology.

[bib77] Hall MR, McGillicuddy E, Kaplan LJ (2014). Biofilm: basic principles, pathophysiology, and implications for clinicians. Surg Infect.

[bib78] Hammar M, Arnqvist A, Bian Z (1995). Expression of two *csg* operons is required for production of fibronectin- and congo red-binding curli polymers in *Escherichia coli* K-12. Mol Microbiol.

[bib79] Hammar M, Bian Z, Normark S (1996). Nucleator-dependent intercellular assembly of adhesive curli organelles in *Escherichia coli*. P Natl Acad Sci USA.

[bib80] Hammer ND, Schmidt JC, Chapman MR (2007). The curli nucleator protein, CsgB, contains an amyloidogenic domain that directs CsgA polymerization. P Natl Acad Sci USA.

[bib81] Hamon MA, Lazazzera BA (2001). The sporulation transcription factor Spo0A is required for biofilm development in *Bacillus subtilis*. Mol Microbiol.

[bib82] Hamon MA, Stanley NR, Britton RA (2004). Identification of AbrB-regulated genes involved in biofilm formation by *Bacillus subtilis*. Mol Microbiol.

[bib83] Hartford OM, Wann ER, Hook M (2001). Identification of residues in the *Staphylococcus aureus* fibrinogen-binding MSCRAMM clumping factor A (ClfA) that are important for ligand binding. J Biol Chem.

[bib84] Hasman H, Chakraborty T, Klemm P (1999). Antigen-43-mediated autoaggregation of *Escherichia coli* is blocked by fimbriation. J Bacteriol.

[bib85] Henderson IR, Meehan M, Owen P (1997). Antigen 43, a phase-variable bipartite outer membrane protein, determines colony morphology and autoaggregation in *Escherichia coli* K-12. FEMS Microbiol Lett.

[bib86] Heras B, Totsika M, Peters KM (2014). The antigen 43 structure reveals a molecular Velcro-like mechanism of autotransporter-mediated bacterial clumping. P Natl Acad Sci USA.

[bib87] Hobley L, Ostrowski A, Rao FV (2013). BslA is a self-assembling bacterial hydrophobin that coats the *Bacillus subtilis* biofilm. P Natl Acad Sci USA.

[bib88] Hollenbeck EC, Fong JCN, Lim JY (2014). Molecular Determinants of mechanical properties of *V. cholerae* biofilms at the air-liquid interface. Biophys J.

[bib89] Hung C, Zhou Y, Pinkner JS (2013). *Escherichia coli* biofilms have an organized and complex extracellular matrix structure. mBio.

[bib90] Hunter S, Jones P, Mitchell A (2012). InterPro in 2011: new developments in the family and domain prediction database. Nucleic Acids Res.

[bib91] Huq A, Xu B, Chowdhury MA (1996). A simple filtration method to remove plankton-associated *Vibrio cholerae* in raw water supplies in developing countries. Appl Environ Microb.

[bib92] Ivleva NP, Wagner M, Horn H (2008). In situ surface-enhanced Raman scattering analysis of biofilm. Anal Chem.

[bib93] Ivleva NP, Wagner M, Szkola A (2010). Label-free in situ SERS imaging of biofilms. J Phys Chem B.

[bib94] James GA, Swogger E, Wolcott R (2008). Biofilms in chronic wounds. Wound Repair Regen.

[bib95] Jansen KU, Girgenti DQ, Scully IL (2013). Vaccine review: ‘*Staphyloccocus aureus* vaccines: problems and prospects’. Vaccine.

[bib96] Johnson M, Zaretskaya I, Raytselis Y (2008). NCBI BLAST: a better web interface. Nucleic Acids Res.

[bib97] Johnson TL, Fong JC, Rule C (2014). The Type II secretion system delivers matrix proteins for biofilm formation by *Vibrio cholerae*. J Bacteriol.

[bib98] Jones SE, Paynich ML, Kearns DB (2014). Protection from intestinal inflammation by bacterial exopolysaccharides. J Immunol.

[bib99] Jones SM, Morgan M, Humphrey TJ (2001). Effect of vancomycin and rifampicin on meticillin-resistant *Staphylococcus aureus* biofilms. Lancet.

[bib100] Kamruzzaman M, Udden SM, Cameron DE (2010). Quorum-regulated biofilms enhance the development of conditionally viable, environmental *Vibrio cholerae*. P Natl Acad Sci USA.

[bib101] Kaper JB, Nataro JP, Mobley HL (2004). Pathogenic *Escherichia coli.* Nature reviews. Microbiology.

[bib102] Khurana R, Uversky VN, Nielsen L (2001). Is Congo red an amyloid-specific dye?. J Biol Chem.

[bib104] Kierek K, Watnick PI (2003a). Environmental determinants of *Vibrio cholerae* biofilm development. Appl Environ Microb.

[bib103] Kierek K, Watnick PI (2003b). The *Vibrio cholerae* O139 O-antigen polysaccharide is essential for Ca2+-dependent biofilm development in sea water. P Natl Acad Sci USA.

[bib105] Kjaergaard K, Schembri MA, Hasman H (2000a). Antigen 43 from *Escherichia coli* induces inter- and intraspecies cell aggregation and changes in colony morphology of *Pseudomonas fluorescens*. J Bacteriol.

[bib106] Kjaergaard K, Schembri MA, Ramos C (2000b). Antigen 43 facilitates formation of multispecies biofilms. Environ Microbiol.

[bib107] Kloepper JW, Ryu CM, Zhang S (2004). Induced systemic resistance and promotion of plant growth by Bacillus spp. Phytopathology.

[bib108] Kobayashi K (2007). *Bacillus subtilis* pellicle formation proceeds through genetically defined morphological changes. J Bacteriol.

[bib109] Kobayashi K, Iwano M (2012). BslA (YuaB) forms a hydrophobic layer on the surface of *Bacillus subtilis* biofilms. Mol Microbiol.

[bib110] Kovacs AT, Kuipers OP (2011). Rok regulates *yuaB* expression during architecturally complex colony development of *Bacillus subtilis* 168. J Bacteriol.

[bib111] Lanni EJ, Masyuko RN, Driscoll CM (2014). MALDI-guided SIMS: multiscale imaging of metabolites in bacterial biofilms. Anal Chem.

[bib112] LeQuere B, Ghigo JM (2009). BcsQ is an essential component of the *Escherichia coli* cellulose biosynthesis apparatus that localizes at the bacterial cell pole. Mol Microbiol.

[bib113] Letunic I, Doerks T, Bork P (2014). SMART: recent updates, new developments and status in 2015. Nucleic Acids Res.

[bib114] Ljungdahl LG, Eriksson K-E, Marshall KC (1985). Ecology of microbial cellulose degradation. Advances in Microbial Ecology.

[bib115] Loferer H, Hammar M, Normark S (1997). Availability of the fibre subunit CsgA and the nucleator protein CsgB during assembly of fibronectin-binding curli is limited by the intracellular concentration of the novel lipoprotein CsgG. Mol Microbiol.

[bib116] Lower SK, Lamlertthon S, Casillas-Ituarte NN (2011). Polymorphisms in fibronectin binding protein A of *Staphylococcus aureus* are associated with infection of cardiovascular devices. P Natl Acad Sci USA.

[bib119] McCrate OA, Zhou X, Reichhardt C (2013). Sum of the parts: composition and architecture of the bacterial extracellular matrix. J Mol Biol.

[bib117] Maira-Litran T, Kropec A, Abeygunawardana C (2002). Immunochemical properties of the staphylococcal poly-N-acetylglucosamine surface polysaccharide. Infect Immun.

[bib118] Marlow VL, Cianfanelli FR, Porter M (2014). The prevalence and origin of exoprotease-producing cells in the *Bacillus subtilis* biofilm. Microbiology.

[bib120] Mehlin C, Headley CM, Klebanoff SJ (1999). An inflammatory polypeptide complex from *Staphylococcus epidermidis:* isolation and characterization. J Exp Med.

[bib121] Merino N, Toledo-Arana A, Vergara-Irigaray M (2009). Protein A-mediated multicellular behavior in *Staphylococcus aureus*. J Bacteriol.

[bib122] Mhatre E, Monterrosa RG, Kovacs AT (2014). From environmental signals to regulators: modulation of biofilm development in Gram-positive bacteria. J Basic Microbiol.

[bib123] Mielich-Suss B, Lopez D (2015). Molecular mechanisms involved in *Bacillus subtilis* biofilm formation. Environ Microbiol.

[bib124] Moorthy S, Watnick PI (2005). Identification of novel stage-specific genetic requirements through whole genome transcription profiling of *Vibrio cholerae* biofilm development. Mol Microbiol.

[bib125] Mulcahy ME, Geoghegan JA, Monk IR (2012). Nasal colonisation by *Staphylococcus aureus* depends upon clumping factor B binding to the squamous epithelial cell envelope protein loricrin. PLoS Pathog.

[bib126] Nagorska K, Bikowski M, Obuchowski M (2007). Multicellular behaviour and production of a wide variety of toxic substances support usage of *Bacillus subtilis* as a powerful biocontrol agent. Acta Biochim Pol.

[bib127] Nenninger AA, Robinson LS, Hammer ND (2011). CsgE is a curli secretion specificity factor that prevents amyloid fibre aggregation. Mol Microbiol.

[bib128] Nenninger AA, Robinson LS, Hultgren SJ (2009). Localized and efficient curli nucleation requires the chaperone-like amyloid assembly protein CsgF. P Natl Acad Sci USA.

[bib129] Neu TR, Lawrence JR (2014). Advanced techniques for in situ analysis of the biofilm matrix (structure, composition, dynamics) by means of laser scanning microscopy. Method Mol Biol.

[bib130] Nichols PD, Henson JM, Guckert JB (1985). Fourier transform-infrared spectroscopic methods for microbial ecology: analysis of bacteria, bacteria-polymer mixtures and biofilms. J Microbiol Method.

[bib131] Nyholm SV, Stabb EV, Ruby EG (2000). Establishment of an animal-bacterial association: recruiting symbiotic vibrios from the environment. P Natl Acad Sci USA.

[bib132] Oli MW, Otoo HN, Crowley PJ (2012). Functional amyloid formation by *Streptococcus mutans*. Microbiology.

[bib133] Olsen A, Jonsson A, Normark S (1989). Fibronectin binding mediated by a novel class of surface organelles on *Escherichia coli*. Nature.

[bib134] Omadjela O, Narahari A, Strumillo J (2013). BcsA and BcsB form the catalytically active core of bacterial cellulose synthase sufficient for in vitro cellulose synthesis. P Natl Acad Sci USA.

[bib135] O'Neill E, Pozzi C, Houston P (2007). Association between methicillin susceptibility and biofilm regulation in *Staphylococcus aureus* isolates from device-related infections. J Clin Microbiol.

[bib136] O'Neill E, Pozzi C, Houston P (2008). A novel *Staphylococcus aureus* biofilm phenotype mediated by the fibronectin-binding proteins, FnBPA and FnBPB. J Bacteriol.

[bib137] Ostrowski A, Mehert A, Prescott A (2011). YuaB functions synergistically with the exopolysaccharide and TasA amyloid fibers to allow biofilm formation by *Bacillus subtilis*. J Bacteriol.

[bib138] Periasamy S, Joo HS, Duong AC (2012). How *Staphylococcus aureus* biofilms develop their characteristic structure. P Natl Acad Sci USA.

[bib139] Pesavento C, Becker G, Sommerfeldt N (2008). Inverse regulatory coordination of motility and curli-mediated adhesion in *Escherichia coli*. Gene Dev.

[bib140] Petersen TN, Brunak S, von Heijne G (2011). SignalP 4.0: discriminating signal peptides from transmembrane regions. Nat Method.

[bib141] Pokrovskaya V, Poloczek J, Little DJ (2013). Functional characterization of *Staphylococcus epidermidis* IcaB, a de-N-acetylase important for biofilm formation. Biochemistry.

[bib142] Prigent-Combaret C, Prensier G, LeThi TT (2000). Developmental pathway for biofilm formation in curli-producing *Escherichia coli* strains: role of flagella, curli and colanic acid. Environ Microbiol.

[bib143] Purdy AE, Watnick PI (2011). Spatially selective colonization of the arthropod intestine through activation of *Vibrio cholerae* biofilm formation. P Natl Acad Sci USA.

[bib144] Queck SY, Jameson-Lee M, Villaruz AE (2008). RNAIII-independent target gene control by the agr quorum-sensing system: insight into the evolution of virulence regulation in *Staphylococcus aureus*. Mol Cell.

[bib145] Reichhardt C, Fong JC, Yildiz F (2015). Characterization of the Vibrio cholerae extracellular matrix: a top-down solid-state NMR approach. Biochim Biophys Acta.

[bib146] Robinson LS, Ashman EM, Hultgren SJ (2006). Secretion of curli fibre subunits is mediated by the outer membrane-localized CsgG protein. Mol Microbiol.

[bib147] Roche FM, Meehan M, Foster TJ (2003). The *Staphylococcus aureus* surface protein SasG and its homologues promote bacterial adherence to human desquamated nasal epithelial cells. Microbiology.

[bib148] Romero D, Aguilar C, Losick R (2010). Amyloid fibers provide structural integrity to *Bacillus subtilis* biofilms. P Natl Acad Sci USA.

[bib149] Romero D, Vlamakis H, Losick R (2011). An accessory protein required for anchoring and assembly of amyloid fibres in *B. subtilis* biofilms. Mol Microbiol.

[bib150] Romero D, Vlamakis H, Losick R (2014). Functional analysis of the accessory protein TapA in *Bacillus subtilis* amyloid fiber assembly. J Bacteriol.

[bib151] Romling U, Bian Z, Hammar M (1998a). Curli fibers are highly conserved between *Salmonella typhimurium* and *Escherichia coli* with respect to operon structure and regulation. J Bacteriol.

[bib152] Romling U, Sierralta WD, Eriksson K (1998b). Multicellular and aggregative behaviour of *Salmonella typhimurium* strains is controlled by mutations in the agfD promoter. Mol Microbiol.

[bib153] Rudrappa T, Quinn WJ, Stanley-Wall NR (2007). A degradation product of the salicylic acid pathway triggers oxidative stress resulting in down-regulation of *Bacillus subtilis* biofilm formation on Arabidopsis thaliana roots. Planta.

[bib154] Schembri MA, Hjerrild L, Gjermansen M (2003). Differential expression of the *Escherichia coli* autoaggregation factor antigen 43. J Bacteriol.

[bib155] Schroeder K, Jularic M, Horsburgh SM (2009). Molecular characterization of a novel *Staphylococcus aureus* surface protein (SasC) involved in cell aggregation and biofilm accumulation. PLoS One.

[bib156] Schultz J, Milpetz F, Bork P (1998). SMART, a simple modular architecture research tool: identification of signaling domains. P Natl Acad Sci USA.

[bib157] Schwartz K, Syed AK, Stephenson RE (2012). Functional amyloids composed of phenol soluble modulins stabilize *Staphylococcus aureus* biofilms. PLoS Pathog.

[bib158] Seminara A, Angelini TE, Wilking JN (2012). Osmotic spreading of *Bacillus subtilis* biofilms driven by an extracellular matrix. P Natl Acad Sci USA.

[bib159] Serra DO, Richter AM, Hengge R (2013a). Cellulose as an architectural element in spatially structured *Escherichia coli* biofilms. J Bacteriol.

[bib160] Serra DO, Richter AM, Klauck G (2013b). Microanatomy at cellular resolution and spatial order of physiological differentiation in a bacterial biofilm. mBio.

[bib161] Sherlock O, Schembri MA, Reisner A (2004). Novel roles for the AIDA adhesin from diarrheagenic *Escherichia coli:* cell aggregation and biofilm formation. J Bacteriol.

[bib162] Sherlock O, Vejborg RM, Klemm P (2005). The TibA adhesin/invasin from enterotoxigenic *Escherichia coli* is self-recognizing and induces bacterial aggregation and biofilm formation. Infect Immun.

[bib163] Sherrard LJ, Tunney MM, Elborn JS (2014). Antimicrobial resistance in the respiratory microbiota of people with cystic fibrosis. Lancet.

[bib164] Shewmaker F, McGlinchey RP, Thurber KR (2009). The functional curli amyloid is not based on in-register parallel beta-sheet structure. J Biol Chem.

[bib165] Shu Q, Crick SL, Pinkner JS (2012). The *E. coli* CsgB nucleator of curli assembles to beta-sheet oligomers that alter the CsgA fibrillization mechanism. P Natl Acad Sci USA.

[bib166] Sjobring U, Pohl G, Olsen A (1994). Plasminogen, absorbed by *Escherichia coli* expressing curli or by Salmonella enteritidis expressing thin aggregative fimbriae, can be activated by simultaneously captured tissue-type plasminogen activator (t-PA). Mol Microbiol.

[bib167] Solano C, Garcia B, Valle J (2002). Genetic analysis of *Salmonella enteritidis* biofilm formation: critical role of cellulose. Mol Microbiol.

[bib168] Stover AG, Driks A (1999a). Control of synthesis and secretion of the *Bacillus subtilis* protein YqxM. J Bacteriol.

[bib169] Stover AG, Driks A (1999b). Regulation of synthesis of the *Bacillus subtilis* transition-phase, spore-associated antibacterial protein TasA. J Bacteriol.

[bib170] Tam NK, Uyen NQ, Hong HA (2006). The intestinal life cycle of *Bacillus subtilis* and close relatives. J Bacteriol.

[bib171] Taylor JD, Zhou Y, Salgado PS (2011). Atomic resolution insights into curli fiber biogenesis. Structure.

[bib172] Terra R, Stanley-Wall NR, Cao G (2012). Identification of *Bacillus subtilis* SipW as a bifunctional signal peptidase that controls surface-adhered biofilm formation. J Bacteriol.

[bib173] Ton-That H, Liu G, Mazmanian SK (1999). Purification and characterization of sortase, the transpeptidase that cleaves surface proteins of *Staphylococcus aureus* at the LPXTG motif. P Natl Acad Sci USA.

[bib174] Ude S, Arnold DL, Moon CD (2006). Biofilm formation and cellulose expression among diverse environmental Pseudomonas isolates. Environ Microbiol.

[bib175] Veening JW, Hamoen LW, Kuipers OP (2005). Phosphatases modulate the bistable sporulation gene expression pattern in *Bacillus subtilis*. Mol Microbiol.

[bib176] Verhamme DT, Murray EJ, Stanley-Wall NR (2009). DegU and Spo0A jointly control transcription of two loci required for complex colony development by *Bacillus subtilis*. J Bacteriol.

[bib177] Vlamakis H, Aguilar C, Losick R (2008). Control of cell fate by the formation of an architecturally complex bacterial community. Gene Dev.

[bib178] Vlamakis H, Chai Y, Beauregard P (2013). Sticking together: building a biofilm the *Bacillus subtilis* way. Nat Rev Microbiol.

[bib179] Wang R, Braughton KR, Kretschmer D (2007). Identification of novel cytolytic peptides as key virulence determinants for community-associated MRSA. Nat Med.

[bib180] Wang R, Khan BA, Cheung GY (2011). *Staphylococcus epidermidis* surfactant peptides promote biofilm maturation and dissemination of biofilm-associated infection in mice. J Clin Invest.

[bib181] Wang X, Hammer ND, Chapman MR (2008). The molecular basis of functional bacterial amyloid polymerization and nucleation. J Biol Chem.

[bib182] Wang X, Preston JF, Romeo T (2004). The *pgaABCD* locus of *Escherichia coli* promotes the synthesis of a polysaccharide adhesin required for biofilm formation. J Bacteriol.

[bib183] Wann ER, Gurusiddappa S, Hook M (2000). The fibronectin-binding MSCRAMM FnbpA of *Staphylococcus aureus* is a bifunctional protein that also binds to fibrinogen. J Biol Chem.

[bib184] White AP, Collinson SK, Banser PA (2001). Structure and characterization of AgfB from *Salmonella enteritidis* thin aggregative fimbriae. J Mol Biol.

[bib185] Wilking JN, Zaburdaev V, De Volder M (2013). Liquid transport facilitated by channels in *Bacillus subtilis* biofilms. P Natl Acad Sci USA.

[bib186] Yaron S, Romling U (2014). Biofilm formation by enteric pathogens and its role in plant colonization and persistence. Microbial Biotechnol.

[bib187] Yildiz F, Fong J, Sadovskaya I (2014). Structural characterization of the extracellular polysaccharide from *Vibrio cholerae* O1 El-Tor. PLoS One.

[bib188] Yildiz FH, Schoolnik GK (1999). *Vibrio cholerae* O1 El Tor: identification of a gene cluster required for the rugose colony type, exopolysacharide production, chlorine resistance, and biofilm formation. P Natl Acad Sci USA.

[bib189] Ziebuhr W, Heilmann C, Gotz F (1997). Detection of the intercellular adhesion gene cluster (ica) and phase variation in *Staphylococcus epidermidis* blood culture strains and mucosal isolates. Infect Immun.

[bib190] Ziebuhr W, Krimmer V, Rachid S (1999). A novel mechanism of phase variation of virulence in *Staphylococcus epidermidis*: evidence for control of the polysaccharide intercellular adhesin synthesis by alternating insertion and excision of the insertion sequence element IS256. Mol Microbiol.

[bib191] Zobell CE, Allen EC (1935). The significance of marine bacteria in the fouling of submerged surfaces. J Bacteriol.

[bib192] Zogaj X, Bokranz W, Nimtz M (2003). Production of cellulose and curli fimbriae by members of the family Enterobacteriaceae isolated from the human gastrointestinal tract. Infect Immun.

[bib193] Zogaj X, Nimtz M, Rohde M (2001). The multicellular morphotypes of *Salmonella typhimurium* and *Escherichia coli* produce cellulose as the second component of the extracellular matrix. Mol Microbiol.

[bib194] Zorraquino V, Garcia B, Latasa C (2013). Coordinated cyclic-di-GMP repression of Salmonella motility through YcgR and cellulose. J Bacteriol.

[bib195] Zuckerman JN, Rombo L, Fisch A (2007). The true burden and risk of cholera: implications for prevention and control. Lancet Infect Dis.

